# Supplementary material for: When can whole-genome SNP heritability be reliably estimated from summary statistics?
Source: bioRxiv. 2026 May 16:2026.05.13.724972. Preprint. [Version 1] doi: 10.64898/2026.05.13.724972 (PMC13192620; doi:10.64898/2026.05.13.724972)
Supplement: Supplement 1 [file NIHPP2026.05.13.724972v1-supplement-1.pdf]

bioRxiv preprint doi: https://doi.org/10.64898/2026.05.13.724972; this version posted May 16, 2026. The copyright holder for this preprint (which was not certified by peer review) is the author/funder, who has granted bioRxiv a license to display the preprint in perpetuity. It is made available under a CC-BY 4.0 International license.

Supplementary: When can Whole-Genome SNP Heritability be reliably estimated from summary statistics?

Benjamin Pham[1,*], Samuel Davenport[2], David Azriel[3], and Armin Schwartzman[1,2]

[1]Halicioğlu Data Science Institute, University of California San Diego
[2]Division of Biostatistics, University of California San Diego
[3]Faculty of Data and Decision Sciences, The Technion, Haifa, Israel
[*]Correspondence: bkpham@ucsd.edu

S1 Corrections to the LD Score Regression Derivation

In this section we seek to clarify the LD score regression derivation. We derive corrected versions of the equations and discuss issues with the original derivation of [8], particularly under population stratification. In order to make this supplementary self-contained we repeat some of the definitions that appear in the main text.

S1.1 Notation and Conditioning

S1.1.1 Notation

We start our derivation by defining our notation, following that of [4]. In particular suppose that we observe data from m SNPs from each of n participants. Define the sample genotype matrix to be $\mathbf{X} \in \mathbb{R}^{n\times m}$ where each row of $\mathbf{X}$ represents the genetic data for a given subject and let $\mathbf{y} \in \mathbb{R}^n$ be a vector of sample phenotypes.

We begin, for simplicity, by considering the model (See Equation (1) from Section 2.1 in the main text) without population stratification. In this case the relationship between sample genotype and phenotype can be represented in a linear model as follows:

$$\mathbf{y} = \mathbf{X}\boldsymbol{\beta} + \boldsymbol{\epsilon} \tag{S1}$$

where $\boldsymbol{\beta} = (\beta_1, \dots, \beta_m)^T \in \mathbb{R}^m$ is the SNP effect size and $\epsilon = (\epsilon_1, \dots, \epsilon_n)^T \in \mathbb{R}^n$ is the error term. For each $1 \le j \le m$, let $\mathbf{X}_j \in \mathbb{R}^n$ be the jth column of $\mathbf{X}$. We make the following model assumptions (as in [4]).

1. For each $1 \le j \le m$, $\mathbf{X}_j$ is normalized to be mean zero and variance one (note that as in [4] we ignore the distinction between normalizing and centering in our sample and in the population)
2. The rows of $\mathbf{X}$ - corresponding to the genotype data for each subject - are independent and identically distributed.
3. $\epsilon_1, \dots, \epsilon_n$ are iid with $\mathrm{E}(\epsilon_i) = 0$ and $\mathrm{var}(\epsilon_i) = 1 - h_g^2$.
4. $\beta_1, \dots, \beta_m$ are iid with $\mathrm{E}(\beta_j) = 0$ and $\mathrm{var}(\beta_j) = \frac{h_g^2}{m}$.
5. $\mathbf{X}, \boldsymbol{\beta}, \boldsymbol{\epsilon}$ are independent.

bioRxiv preprint doi: https://doi.org/10.64898/2026.05.13.724972; this version posted May 16, 2026. The copyright holder for this preprint (which was not certified by peer review) is the author/funder, who has granted bioRxiv a license to display the preprint in perpetuity. It is made available under a CC-BY 4.0 International license.

Define the estimated effect-size of the jth SNP $\hat{\beta}_j$ as:

$$\hat{\beta}_j = \frac{\mathbf{X}_j^T \mathbf{y}}{n}. \tag{S2}$$

Define χ_j^2 as:

$$\chi_j^2 = n\hat{\beta}_j^2 = \frac{\left(\mathbf{X}_j^T \mathbf{y}\right)^2}{n}. \tag{S3}$$

We also define the LD scores as follows. Let $\hat{\mathbf{R}} = \frac{1}{n}\mathbf{X}^T\mathbf{X}$ denote the correlation matrix with entries $\hat{r}_j k$. Then the sample LD scores are computed as:

$$\hat{\ell}_j := \sum_{k=1}^{m} \hat{r}_{jk}^2 = \frac{1}{n^2}\mathbf{X}_j^T\mathbf{X}\mathbf{X}^T\mathbf{X}_j, \qquad 1 \le j \le m \tag{S4}$$

Letting $r_{jk} = \text{cov}(X_{1j}, X_{1k})$, population LD scores can be also defined as

$$\ell_j = \sum_{k=1}^{m} r_{jk}^2, \qquad 1 \le j \le m. \tag{S5}$$

S1.1.2 Conditioning

Before continuing with our explanation of the LD score derivation we first make an observation regarding conditioning which in our view is approached incorrectly in [3]. The paper as a whole targets the unconditional expectation: $\text{E}\left(\chi_j^2\right)$ instead of the conditional expectation $\text{E}\left(\chi_j^2 \middle| X\right)$ (see for instance the statements of Propositions 1 and 2 in [4]). This is problematic and means that the derivation of their estimator without population stratification is circular (as we demonstrate in Section S1.2). Furthermore it causes their derivation under population stratification to be incorrect, as we show in Section S1.3. This in turn means that using the original LD score regression equations leads to bias under population stratification, as we demonstrated in AR1 and Realistic LD simulations from Section 3 in the main text.

The reason that $\text{E}\left(\chi_j^2 \middle| \mathbf{X}\right)$ should be targeted rather than the unconditional expectation is that the summary statistics $(\chi_j^2)_{j=1}^m$ are calculated based on the sample $\mathbf{X}$. There is thus not enough information in the system to infer on $\text{E}\left(\chi_j^2\right)$ as that would require many samples of χ_j^2 from different $\mathbf{X}$ matrices. Furthermore, regression that is based on the unconditional expectation leads to bias under population stratification as explained below in Section S1.3.1.

S1.2 LDSC derivation without population stratification

S1.2.1 LDSC Derivation

In what follows we re-derive the LDSC regression equation with the correct conditioning resulting in Theorem S1.1. This result is the analogue of Proposition 1 of [4], with conditioning correctly accounted for.

Theorem S1.1. *Under Model* (S1),

$$\text{E}\left(\chi_j^2 \,\middle|\, \mathbf{X}\right) = h_g^2\left(\frac{n}{m}\hat{\ell}_j - 1\right) + 1. \tag{S6}$$

Proof. We first note that

$$\text{E}\left(\hat{\beta}_j \,\middle|\, \mathbf{X}\right) = \text{E}\left(\frac{\mathbf{X}_j^T\mathbf{y}}{n} \,\middle|\, \mathbf{X}\right) = \text{E}\left(\frac{\mathbf{X}_j^T\mathbf{X}\boldsymbol{\beta} + \mathbf{X}_j^T\boldsymbol{\epsilon}}{n} \,\middle|\, \mathbf{X}\right) = 0,$$

bioRxiv preprint doi: https://doi.org/10.64898/2026.05.13.724972; this version posted May 16, 2026. The copyright holder for this preprint (which was not certified by peer review) is the author/funder, who has granted bioRxiv a license to display the preprint in perpetuity. It is made available under a CC-BY 4.0 International license.

since $\mathrm{E}(\boldsymbol{\beta}) = \mathbf{0}$ and $\mathrm{E}(\boldsymbol{\epsilon}|\mathbf{X}) = \mathbf{0}$. As such,

$$\begin{aligned}
\mathrm{E}\left(\chi_j^2 \,\middle|\, \mathbf{X}\right) &= n\mathrm{E}\left(\hat{\beta}_j^2 \,\middle|\, \mathbf{X}\right) \\
&= n\left(\mathrm{var}\left(\hat{\beta}_j \,\middle|\, \mathbf{X}\right) + \mathrm{E}\left(\hat{\beta}_j \,\middle|\, \mathbf{X}\right)^2\right) \\
&= n\mathrm{var}\left(\hat{\beta}_j \,\middle|\, \mathbf{X}\right).
\end{aligned}$$

Now,

$$\begin{aligned}
n\mathrm{var}\left(\hat{\beta}_j \,\middle|\, \mathbf{X}\right) &= \frac{1}{n}\mathrm{var}\left(\mathbf{X}_j^T\mathbf{y} \,\middle|\, \mathbf{X}\right) \\
&= \frac{1}{n}\left(\mathbf{X}_j^T\mathrm{var}\left(\mathbf{y} \,|\, \mathbf{X}\right)\mathbf{X}_j\right) \\
&= \frac{1}{n}\left(\mathbf{X}_j^T\left(\frac{h_g^2}{m}\mathbf{X}\mathbf{X}^T + \left(1 - h_g^2\right)I\right)\mathbf{X}_j\right) \\
&= \frac{1}{n}\left(\frac{h_g^2}{m}\mathbf{X}_j^T\mathbf{X}\mathbf{X}^T\mathbf{X}_j + n(1 - h_g^2)\right) \\
&= \frac{h_g^2}{m}\hat{\ell}_j n - h_g^2 n + 1 \\
&= h_g^2\left(\frac{n}{m}\hat{\ell}_j - 1\right) + 1.
\end{aligned}$$

□

The proof follows the begining of Proposition 1 of [4]. However the end result is is exact rather than an approximation. The authors use an approximation of the Olkin and Pratt estimator for population squared correlation [13] combined with a δ method approximation in order to estimate the unconditional expectation. In particular they derive the following unconditional approximation.

$$\mathrm{E}\left(\chi_j^2\right) \approx \frac{nh_g^2}{m}\ell_j + 1. \tag{S7}$$

However, as we show in the next section, using the unadjusted LD scores in (S7) (as in done in practice in the implementation of [3]) actually recovers (S6) - meaning that the use of the approximation and (S7) is unnecessary.

S1.2.2 The circular use of approximations

The LD score equation derived in [4] (displayed in (S7)), depends on the population LD scores which are unknown. In order to get around this [3] propose taking

$$\tilde{r}_{jk}^2 = \hat{r}_{jk}^2 - \frac{1 - \hat{r}_{jk}^2}{n - 2}$$

as this is an unbiased estimator of r_{jk}^2. They then use these to compute adjusted LD scores,

$$\tilde{\ell}_j = \sum_{k=1}^{m}\tilde{r}_{jk}^2, \qquad 1 \leq j \leq m.$$

It follows that

$$\tilde{\ell}_j = \sum_{k=1}^{m}\left\{\hat{r}_{jk}^2 - \frac{1 - \hat{r}_{jk}^2}{n - 2}\right\} = \sum_{k=1}^{m}\hat{r}_{jk}^2 - \sum_{k=1}^{m}\frac{1 - \hat{r}_{jk}^2}{n - 2} = \hat{\ell}_j - \left(\frac{m - \hat{\ell}_j}{n - 2}\right). \tag{S8}$$

bioRxiv preprint doi: https://doi.org/10.64898/2026.05.13.724972; this version posted May 16, 2026. The copyright holder for this preprint (which was not certified by peer review) is the author/funder, who has granted bioRxiv a license to display the preprint in perpetuity. It is made available under a CC-BY 4.0 International license.

In the LDSC software these adjusted LD scores are then plugged into (S7) and used when performing LD score regression. As such, when $\tilde{\ell}_j$ is plugged into the right hand side of (S7) in place of ℓ_j we obtain

$$\begin{aligned}\frac{nh_g^2}{m}\tilde{\ell}_j + 1 &= \frac{nh_g^2}{m}\left(\hat{\ell}_j - \left(\frac{m-\hat{\ell}_j}{n-2}\right)\right) + 1\\ &= h_g^2\left(\frac{n}{m}\hat{\ell}_j - \frac{n}{n-2} + \frac{n\hat{l}_j}{m(n-2)}\right) + 1\\ &= h_g^2\left(\frac{n}{m}\hat{\ell}_j - 1\right) + 1 + \left(1 - \frac{n}{n-2}\right) + \frac{n\hat{\ell}_j h_g^2}{m(n-2)}\\ &= h_g^2\left(\frac{n}{m}\hat{\ell}_j - 1\right) + 1 + O\left(\frac{1}{n}\right) + O\left(\frac{1}{m}\right)\end{aligned}$$

so we recover the right hand side of (S6) up to terms of a low order. This explains why using the unconditional equation, as done in [3] is okay in this case - since in practice the conditional equation is recovered.

Remark S1.2. *The original proof of Proposition 1 of [4] starts off as in the proof of Theorem S1.1 then uses an approximation to derive* (S7) *and then uses a second approximation to estimate the LD scores. As we have shown doing all of this in fact returns us to the result of Theorem S1.1 and so the combination of the two approximations is circular. Since the conditional expression is the one that should be used, as we argued in Section S1.1.2, using these approximations are unnecessary.*

Remark S1.3. *In this setting, using and then undoing the approximation recovers the conditional equation. As such in this simple setting where there is no population stratification the original formula is thus conditionally correct. However, as we shall show in Section S1.3, under the presence of population stratification the equation changes. Unfortunately this means that using the original formula can lead to bias, as demonstrated in Section 3 of the main text.*

S1.3 LDSC regression derivation under Population Stratification

S1.3.1 Modeling Population Stratification

To account for population stratification we follow the model of [4]. We assume that each subject i belongs with equal probability to one of two populations P_1, P_2, and

$$\mathbf{y} = \mathbf{X}\boldsymbol{\beta} + \boldsymbol{S} + \boldsymbol{\epsilon}, \tag{S9}$$

where $S_i|i \in P_1 = \sigma_s$ and $S_i|i \in P_2 = -\sigma_s$ for a constant $\sigma_s > 0$, $\mathrm{E}(X_{ij}|i \in P_1, \mathbf{f}) = \frac{f_j}{\sqrt{1+f_j^2}}$ and $\mathrm{E}(X_{ij}|\mathbf{f}, i \in P_2) = \frac{-f_j}{\sqrt{1+f_j^2}}$, and $\mathrm{var}(X_{ij}|\mathbf{f}) = \frac{1}{1+f_j^2}$, where the vector $\mathbf{f} \in \mathcal{R}^m$ is iid with mean 0 and variance F_{ST}^2. Instead of $\mathrm{var}(\epsilon_i) = 1 - h_g^2$ of Assumption 4 of Model (S1) we now assume that $\mathrm{var}(\epsilon_i) = 1 - h_g^2 - \sigma_s^2$ in order to keep $\mathrm{var}(y_i)$ to be 1. The rest of the Assumptions of Model (S1) holds also here.

S1.3.2 A comment about the definition of S

The population stratification term $\mathbf{S}$ was originally defined in [4] as $S_i|i \in P_1 = \frac{\sigma_s}{2}$ and $S_i|i \in P_2 = -\frac{\sigma_s}{2}$, while in our definition we do not divide by 2. On the other hand, they assumed, as we do, that $\mathrm{var}(\epsilon_i) = 1-h_g^2-\sigma_s^2$, which implicitly requires that $\mathrm{var}(S_i) = \sigma_s^2$ because $\mathrm{var}(y_i) = 1$. However, according to their definition, $\mathrm{var}(S_i) = \frac{\sigma_s^2}{4}$, which makes their definitions inconsistent. To fix this mistake we define S_i as $\pm\sigma_s$ without dividing by 2.

S1.3.3 Varying σ_s in S does not change heritability estimation on average in even subpopulations

Varying σ_s does not change heritability estimator behavior on average in our simulations because there are an equal number of subjects in P_1 and P_2. This shifts $\mathbf{y}$ by $\pm\sigma_s$ over all i individuals which aggregates as 0:

bioRxiv preprint doi: https://doi.org/10.64898/2026.05.13.724972; this version posted May 16, 2026. The copyright holder for this preprint (which was not certified by peer review) is the author/funder, who has granted bioRxiv a license to display the preprint in perpetuity. It is made available under a CC-BY 4.0 International license.

$$
\begin{aligned}
\mathrm{E}(\mathbf{y}) &= \frac{1}{2}\mathrm{E}(\mathbf{y}|P_1) + \frac{1}{2}\mathrm{E}(\mathbf{y}|P_2) \\
&= \frac{1}{2}\mathrm{E}(\mathbf{X}\boldsymbol{\beta} + \mathbf{S} + \boldsymbol{\epsilon}|P_1) + \frac{1}{2}\mathrm{E}(\mathbf{X}\boldsymbol{\beta} + \mathbf{S} + \boldsymbol{\epsilon}|P_2) \\
&= \frac{1}{2}\mathrm{E}(\mathbf{X}\boldsymbol{\beta}) + \frac{1}{2}\sigma_s\mathbf{1} + \frac{1}{2}\mathrm{E}(\mathbf{X}\boldsymbol{\beta}) - \frac{1}{2}\sigma_s\mathbf{1} \\
&= \mathrm{E}(\mathbf{X}\boldsymbol{\beta}).
\end{aligned}
$$

S1.3.4 A mistake in the derivation of [4]

The derivation [4] contains a critical mistake, which, as explained below and demonstrated in Sections 3.1 and 3.2 of the main text, causes their estimate to be severely biased when σ_s and F_{ST}^2 is large. In [4] on page 4 it is written that "we compute $\mathrm{E}[\chi_j^2]$ with the expectation taken over random $\mathbf{X}$, $\boldsymbol{\beta}$, $\boldsymbol{\epsilon}$, $\mathbf{f}$ but with $\mathbf{S}$ fixed to ensure population stratification." However, Eq. (2.11) of [4] reads $\mathrm{E}[\hat{\beta}_j|\mathbf{X}] = \frac{1}{N}\mathbf{X}_j^T\mathbf{S} = f\sigma_s$ (in our notation $n = N$); the first equality is correct, but the second is wrong as $\mathbf{X}$ is conditioned upon and $\mathbf{S}$ is fixed. Hence, $\mathrm{E}[\hat{\beta}_j|\mathbf{X}] = \frac{1}{N}\mathbf{X}_j^T\mathbf{S}$. As we shall see below, this mistake results in bias of the suggested estimator of [4].

S1.3.5 The conditional expectation of χ_j^2 and the resulting bias of the estimator

The equivalent result of Theorem S1.1 under Model (S9) is given now. To make the argument clear, the conditioning on $\mathbf{S}$ is explicit in the notation.

Theorem S1.4. *Under Model* (S9),

$$
\mathrm{E}\left(\chi_j^2 \,\middle|\, \mathbf{X}, \mathbf{S}\right) = h_g^2\left(\frac{n}{m}\ell_j - 1\right) + 1 - \sigma_s^2 + \frac{1}{n}\left(\mathbf{X}_j^T\mathbf{S}\right)^2. \tag{S10}
$$

Proof. We proceed as in the proof of Theorem S1.1. As mentioned in Section S1.3.4,

$$
\mathrm{E}\left(\hat{\beta}_j|\mathbf{X}, \mathbf{S}\right) = \frac{1}{n}\mathbf{X}_j^T\mathbf{S}.
$$

The computation of $n\mathrm{var}\left(\hat{\beta}_j \,\middle|\, \mathbf{X}, \mathbf{S}\right)$ is almost the same as in the proof of Theorem S1.1, besides that now $\mathrm{var}(\epsilon_i) = 1 - h_g^2 - \sigma_s^2$ (while under Model (S1), $\mathrm{var}(\epsilon_i) = 1 - h_g^2$). It follows that

$$
n\mathrm{var}\left(\hat{\beta}_j \,\middle|\, \mathbf{X}, \mathbf{S}\right) = h_g^2\left(\frac{n}{m}\hat{\ell}_j - 1\right) + 1 - \sigma_s^2.
$$

Putting the terms together in

$$
\mathrm{E}\left(\chi_j^2 \,\middle|\, \mathbf{X}, \mathbf{S}\right) = n\left(\mathrm{var}\left(\hat{\beta}_j \,\middle|\, \mathbf{X}, \mathbf{S}\right) + \mathrm{E}\left(\hat{\beta}_j \,\middle|\, \mathbf{X}, \mathbf{S}\right)^2\right),
$$

yields (S10). □

The key term in the conditional expectation (S10) is the last term $\frac{1}{n}\left(\mathbf{X}_j^T\mathbf{S}\right)^2$, which was not calculated properly in [4] as mentioned earlier. This term is correlated with $\hat{\ell}_j$, which leads to bias estimator as explained next.

Theorem S1.4 demonstrates a conceptual mistake in the derivation of [4]. As mentioned above they calculate the unconditional expectation of $\mathrm{E}\left(\chi_j^2\right)$. However writing,

$$
\chi_j^2 = \mathrm{E}\left(\chi_j^2\right) + \text{error term},
$$

Theorem S1.4 implies that the error term is correlated with $\hat{\ell}_j$. Therefore, when $\{\chi_j^2\}_{j=1}^m$ is regressed on the LD-scores $\{\hat{\ell}_j\}_{j=1}^m$, the resulting estimator will be biased due to this correlation. On the other hand, when one considers the conditional expectation $\mathrm{E}\left(\chi_j^2 \,\middle|\, \mathbf{X}, \mathbf{S}\right)$ the error term is not correlated with $\mathbf{X}$ and $\mathbf{S}$ by definition. Thus, one should base the regression estimator on the conditional expectation rather the unconditional one - not doing so can lead to bias.

bioRxiv preprint doi: https://doi.org/10.64898/2026.05.13.724972; this version posted May 16, 2026. The copyright holder for this preprint (which was not certified by peer review) is the author/funder, who has granted bioRxiv a license to display the preprint in perpetuity. It is made available under a CC-BY 4.0 International license.

Remark S1.5. *As we observed in Section 3 and Figures 1 and 2 from the main text, LDSC is biased under population stratification despite [3] claiming that adding the bias correction term should resolve this. We have now explained this as an issue with the derivation and fact that the error is correlated with the LD scores. The bias of the LDSC estimator is calculated explicitly in Theorem 9 of [1], where it is shown that it is asymptotically equal to* $\sigma_s^2/\mathrm{E}[f_j^2/(1+f_j^2)]$. *Note that this issue does not affect the model without population stratification because the error in the conditional expectation* (S6) *is not correlated with the LD scores (and indeed because as argued in Section S1.2.2 the actual implementation of the LDSC regression estimator with the fixed intercept reduces to the conditional version).*

bioRxiv preprint doi: https://doi.org/10.64898/2026.05.13.724972; this version posted May 16, 2026. The copyright holder for this preprint (which was not certified by peer review) is the author/funder, who has granted bioRxiv a license to display the preprint in perpetuity. It is made available under a CC-BY 4.0 International license.

S2 Implementation Details

S2.1 LDSC Implementation

S2.1.1 LDSC Equation in Practice

The current implementation of LDSC is built with functional annotations in mind [7]. While m in Equation (8) in the main text is meant to be interpreted as a scalar value of all SNPs in the summary statistic, M in the current release of the LDSC code is actually a $c \times 1$ vector which sums to m where c is the number of functional annotations and each element of M is the number of SNPs per c annotations. Each SNP can have different sample sizes due to genotyping or quality control [9]. This is represented as N, a $m \times 1$ vector of sample-size per genotyped SNP. LDSC uses the average of N as n in Equation (8).

S2.1.2 LDSC Method Overview

LD Score Regression heritability is computed in two steps (three steps in actuality). For this explanation, we assume that $\tilde{\ell}_j$ is observed. If not, then it can be substituted with $\tilde{\ell}_j^*$ as described in the main text.

0. A "crude" estimate of heritability is calculated by a ratio of means: The mean of χ_j^2s $\left(\overline{\chi^2}\right)$ on the mean of LD scores $\left(\bar{\tilde{\ell}}\right)$. This is known as the "aggregate" heritability estimate and is given by the following expression:

$$h^2_{\text{crude}} = \frac{m\left(\overline{\chi^2} - 1\right)}{n\left(\bar{\tilde{\ell}}\right)}$$

   This heritability estimate is susceptible to population stratification effects and is used to compute initial weights.

   The weighting stage consists of the following:

   (a) Heritability is bound between 0 or 1.

   (b) The minimum value ℓ_j can take is 1 and is set to 1 if below it.

   (c) Initial weights are computed:

$$w_j = \frac{1}{2(\text{intercept} + h^2 \frac{n}{m} * \tilde{\ell}_j)^2} \circ \frac{1}{\ell_j^w}$$

   Where $\tilde{\ell}_j$ represents the LD scores of specific SNPs of interest. ℓ_j^w are LD scores of the same SNPs from potentially another trusted panel (such as HapMap3 SNP LD scores). When the intercept is not yet estimated, intercept $= 1$ in w_j. In LDSC, weighting is done for the following reasons:

   i. The HapMap "regression SNPs" LD scores come from LD scores computed from JUST HapMap3 SNPs. This is because χ^2 statistics used in the regression are not independent.

   ii. Heteroskedasity in SNPs meaning that χ^2 of SNPs with high LD have higher variance than χ^2 if SNPs with lower LD so SNPs with high LD scores are down-weighted.

   In our scenarios, we do not explore the inclusion of a trusted reference panel and we use all SNPs so $\tilde{\ell}_j = \ell_j^w$. Further, LDSC "is not sensitive to the precise choice of" ℓ_j^w [6].

1. Starting with the inital weights, iterative weighted least squares is performed on SNPs with $\chi_j^2 <$ twostep (default: twostep $= 30$).

   (a) Do weighted least squares by weighting $\tilde{\ell}_j$ by w_j and χ_j^2 by w_j and fitting the regression. In this step, $\frac{n}{m}h^2$ and an intercept is estimated.

   (b) From the weighted least squares results, the new h^2 and intercept are used to compute new weights. This occurs twice.

bioRxiv preprint doi: https://doi.org/10.64898/2026.05.13.724972; this version posted May 16, 2026. The copyright holder for this preprint (which was not certified by peer review) is the author/funder, who has granted bioRxiv a license to display the preprint in perpetuity. It is made available under a CC-BY 4.0 International license.

(c) The $\tilde{\ell}_j$ and χ_j^2 are weighted by the final weights from the above step and a final weighted least squares regression is fit.

2. Using the intercept from the previous step, do iterative weighted least squares on the whole dataset ($\tilde{\ell}_j$ and χ_j^2 unfiltered) with the starting initial weights (steps a through c in step 1).

From the results of the iterative weighted least squares, the "jackknife process" is conducted. Jackknife is usually used to get standard errors by leaving-one-out. The implementation outputs the estimate during this process as well. We follow typical linear regression notation where $\mathtt{X}$ represents the design matrix and $\mathtt{y}$ represents the response, not genotype and phenotype respectively. In a very simple case, $\mathtt{X}$ is a $m \times 2$ matrix with a column of ones and $\tilde{\ell}_j$s and $\mathtt{y}$ is an $m \times 1$ vector of χ_j^2s. The following steps occur:

1. Blocks (default: 200) of $\mathtt{X}$ and $\mathtt{y}$ are created. For each kth block, calculate $\left(\mathtt{X}^T\mathtt{X}\right)_k$ and $\left(\mathtt{X}^T\mathtt{y}\right)_k$.

2. Sum across all $\left(\mathtt{X}^T\mathtt{X}\right)_k$s and $\left(\mathtt{X}^T\mathtt{y}\right)_k$ s to make total $\mathtt{X}^T\mathtt{X}$ and total $\mathtt{X}^T\mathtt{y}$. Calculate $\frac{N}{M}h^2$ from the OLS Normal Equation:
$$\hat{\beta} = \left(\mathtt{X}^T\mathtt{X}\right)^{-1}\mathtt{X}^T\mathtt{y}.$$

3. Create delete values. The kth delete value $\left(\hat{\beta}_{k,del}\right)$ is the $\hat{\beta}$ calculated after subtracting the jth $\mathtt{X}^T\mathtt{X}$ from the total $\mathtt{X}^T\mathtt{X}$ and subtracting the kth $\mathtt{X}^T\mathtt{y}$ from the total $\mathtt{X}^T\mathtt{y}$.

4. Create pseudovalues. The kth pseudovalue (P) is the impact of removing the kth block from the total calculations:
$$P_k = n * \hat{\beta} - (n-1) * \hat{\beta}_{k,del}.$$

The Pseudovalue matrix ($\mathbf{P}$) is a $k \times 2$ matrix since two estimates are computed: the intercept and the heritability estimate. The covariance matrix is computed as:

$$\frac{1}{n}\mathbf{P}^T\mathbf{P}$$

and the diagonals are the block-jackknifed variance of each parameter. The reported standard error in the LDSC output is the square-root of the diagonals.

S2.1.3 LD Score Comparisons

We constructed LD scores from 1000 Genomes Phase I data as described in [3] directly using the LDSC package. The individual that was excluded "from a pair of cousins" in [3] was not identified so we created LD scores of chromosome 22 where each individual was excluded iteratively and compared the output against the released LD scores of chromosome 22 via MSE. The individuals in these LD scores with the lowest MSE of 0.003844 were used to construct the LD scores for the remaining chromosomes. The heritability estimates from our constructed LD scores with the lowest MSE closely matches those estimated with the released LD scores.

S2.1.4 Effect of the twostep Parameter in LDSC

The intercept is first estimated by fitting a regression with SNPs that have $\chi_j^2 \leq$ twostep, where twostep is a predetermined threshold. The rationale for this is that SNPs with small χ_j^2 under this threshold are less likely to be causal [2]. The twostep parameter is set by default in LDSC as 30.

bioRxiv preprint doi: https://doi.org/10.64898/2026.05.13.724972; this version posted May 16, 2026. The copyright holder for this preprint (which was not certified by peer review) is the author/funder, who has granted bioRxiv a license to display the preprint in perpetuity. It is made available under a CC-BY 4.0 International license.

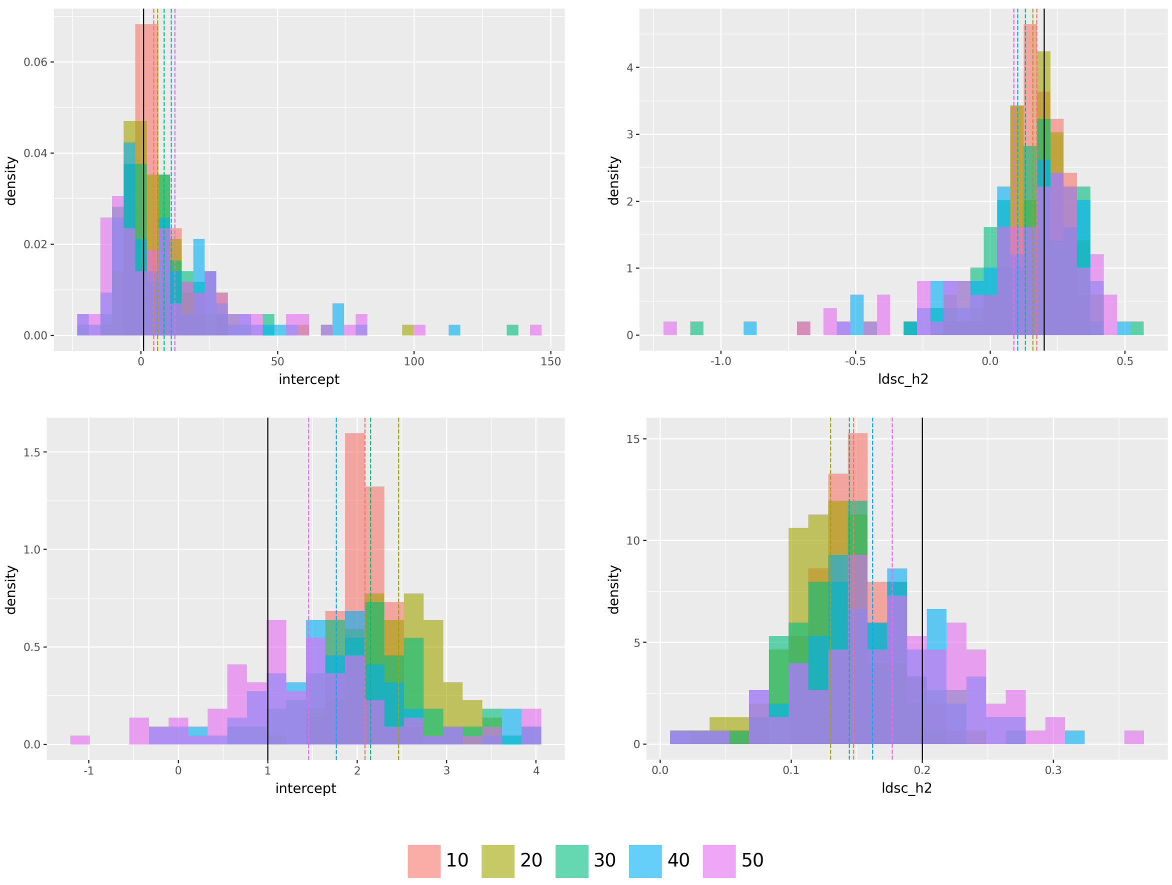

Figure S1: Histograms of LDSC estimated intercepts and $\hat{h}^2$ in AR1 $\rho = 0.995$ (top row) and Realistic (bottom row) settings of 100 simulations with varying twostep (colors) and no population stratification. The black line in the intercept histogram represents an intercept of 1 and the black line in the heritability histogram represents $h_g^2 = 0.2$. The dashed lines are the average quantity across the 100 simulation replicates. Note that the density scale across the histograms are different.

In the AR1 setting, increasing twostep results in a larger intercept on average. In the realistic setting, a large twostep results in a smaller intercept. The intercept is estimated to be larger than 1 under the default setting of twostep $= 30$ resulting in a downwards bias of $\hat{h}^2$ in both settings. Increasing the twostep parameter in the AR1 setting increases the intercept away from 1 and biases the heritability estimate downwards away from $h_g^2 = 0.2$, on average. Unlike the AR1 setting, a clear trend is not present in the realistic setting with respect to the twostep parameter. We do note that a twostep $= 50$ results in an intercept closest to 1 and a heritability estimate closest to $h_g^2 = 0.2$, on average.

S2.2 GCTA Implementation

We compare our implementation (Python GCTA) to the released implementation (Native GCTA) from [12]. We measured the computation time of each algorithm and its heritability estimate across 10 simulations with $n = 5000$, $m = 10000$ 1000G chr 22 SNP correlation. On an Intel computational platform, the computation time is comparable:

Implementation	$\hat{h}^2$	Iterations to Converge	Computation Time (s)
Native GCTA	0.1950 (0.0279)	10.000 (0)	51.4160 (0.6205)
Python GCTA	0.1950 (0.0279)	8.6000 (0.5477)	66.6420 (4.2146)

bioRxiv preprint doi: https://doi.org/10.64898/2026.05.13.724972; this version posted May 16, 2026. The copyright holder for this preprint (which was not certified by peer review) is the author/funder, who has granted bioRxiv a license to display the preprint in perpetuity. It is made available under a CC-BY 4.0 International license.

On an AMD platform, the time needed for the Python implementation is ~ 2.7 times worse:

Implementation	$\hat{h}^2$	Iterations to Converge	Computation Time (s)
Native GCTA	0.1950 (0.0279)	10.0000 (0)	39.5220 (0.2372)
Python GCTA	0.1950 (0.0279)	8.6000 (0.5477)	107.6062 (10.7435)

We saw that despite having heritability estimates that are really close $\sim 10^{-7}$, there is a difference in computation time between the two implementations favoring Native GCTA. The advantage of using the Python GCTA implementation is that it integrates seamlessly in our framework, and that we do not need to consider computational overhead of writing/formating the simulated data as typical input files for Native GCTA (ie: GRM, .pheno files) and reading on the spot.

S2.2.1 GCTA Algorithm

The inputs for this are:

1. y, the phenotype individual-level data, an $n \times 1$ vector.
2. $\mathbf{K} = \mathbf{X}\mathbf{X}^T$, the kinship matrix (also called the GRM in [12]), an $n \times n$ matrix constructed from individual-level genetic data $\mathbf{X}$.

The GCTA algorithm estimates σ_g^2 and σ_e^2 iteratively using the Expectation Maximization (EM) algorithm and Average Information (AI) algorithm. The EM algorithm as the initial step to determine the direction of iteration updates and AI algorithm for the remaining steps until convergence [12]. The original implementation of GCTA considers multiple variance components per region of interest (ie: chromosomes) [12], but for our study we decided to look at only one region. Therefore, our scheme here only considers 2 variance components whereas the original paper has a generalized derivation for multiple variance components.

We start with the initial EM algorithm in the following steps:

1. Start with guesses: $\sigma_g^2 = \sigma_e^2 = \frac{1}{2}\frac{\sum_{i=1}^{n}(y_i-\bar{y})^2}{n-1}$.
2. Construct $\mathbf{V} = \sigma_g^2\mathbf{K} + \sigma_e^2\mathbf{I}$. This is an $n \times n$ matrix.
3. Construct projection matrix $\mathbf{P} = \mathbf{V}^{-1} - (\mathbf{V}^{-1}\mathbf{A})\mathbf{A}^T\mathbf{V}^{-1}\mathbf{A}^{-1}\mathbf{A}^T\mathbf{V}^{-1}$. This is an $n \times n$ matrix. The original GCTA implementation examines c non-genetic covariates for each n subject. This is represented as $\mathbf{A}$, a $n \times c$ matrix. Since we do not examine these in this work, we omit explicit mention from the main text and this is just an intercept (ie: a column of ones).
4. Calculate the log-likelihood (L) given $\mathbf{V}$ and $\mathbf{y}$. This is:

$$L(\mathbf{V}, \mathbf{y}) = -0.5 * (|\mathbf{V}| + |\mathbf{A}^T\mathbf{V}^{-1}\mathbf{A}| + \mathbf{y}^T\mathbf{P}\mathbf{y})$$

   where $|\mathbf{V}|$ is the determinant of $\mathbf{V}$ and the other symbols are defined as before.

5. Compute the initial update of σ_g^2 and σ_e^2.

$$(\sigma_g^2)^{(1)} = \frac{1}{n}(\sigma_g^2\mathbf{y}^T\mathbf{P}\mathbf{K}\mathbf{P}\mathbf{y} + \text{tr}(\sigma_g^2\mathbf{I}_n - \sigma_g^2\mathbf{P}\mathbf{K}))$$
$$(\sigma_e^2)^{(1)} = \frac{1}{n}(\sigma_e^2\mathbf{y}^T\mathbf{P}\mathbf{P}\mathbf{y} + \text{tr}(\sigma_e^2\mathbf{I}_n - \sigma_e^2\mathbf{P}))$$

The σ_g^2 and σ_e^2 from the EM algorithm are used as the starting points for the AI algorithm. The AI algorithm runs until abs $\left((L(\mathbf{V}, y))^{(t)} - (L(\mathbf{V}, y))^{(t-1)}\right) < 10^{-4}$. For each t iteration:

1. Check if $(\sigma_g^2)^{(t)} < 0$. If it is, then set $(\sigma_g^2)^{(t)} = (\frac{\sum_{i=1}^{n}(y_i-\bar{y})^2}{n-1}) * 10^{-6}$
2. Check if $(\sigma_e^2)^{(t)} < 0$. If it is, then set $(\sigma_e^2)^{(t)} = (\frac{\sum_{i=1}^{n}(y_i-\bar{y})^2}{n-1}) * 10^{-6}$

bioRxiv preprint doi: https://doi.org/10.64898/2026.05.13.724972; this version posted May 16, 2026. The copyright holder for this preprint (which was not certified by peer review) is the author/funder, who has granted bioRxiv a license to display the preprint in perpetuity. It is made available under a CC-BY 4.0 International license.

3. Compute $(\mathbf{V})^{(t)} = (\sigma_g^2)^{(t)}\mathbf{K} + (\sigma_e^2)^{(t)}\mathbf{I}$
4. Compute $(\mathbf{P})^{(t)} = (\mathbf{V}^{-1})^{(t)} - ((\mathbf{V}^{-1})^{(t)}\mathbf{A})\mathbf{A}^T(\mathbf{V}^{-1})^{(t)}\mathbf{A}^{-1}\mathbf{A}^T(\mathbf{V}^{-1})^{(t)}$.
5. Compute $L\left(\mathbf{V}, y\right))^{(t)} = -0.5 * (|(\mathbf{V})^{(t)}| + |\mathbf{A}^T(\mathbf{V}^{-1})^{(t)}\mathbf{A}| + y^T(\mathbf{P})^{(t)}y)$.
6. Check if abs $\left((L\left(\mathbf{V}, y\right))^{(t)} - (L\left(\mathbf{V}, y\right))^{(t-1)}\right) < 10^{-4}$. If this statement is true, stop; otherwise, go to the next steps.
7. Compute AI matrix:
$$\mathbf{Q} = \frac{1}{2}\begin{bmatrix} y^T(\mathbf{P})^{(t)}\mathbf{K}(\mathbf{P})^{(t)}\mathbf{K}(\mathbf{P})^{(t)}y & y^T(\mathbf{P})^{(t)}\mathbf{K}(\mathbf{P})^{(t)}(\mathbf{P})^{(t)}y \\ y^T(\mathbf{P})^{(t)}(\mathbf{P})^{(t)}\mathbf{K}(\mathbf{P})^{(t)}y & y^T(\mathbf{P})^{(t)}(\mathbf{P})^{(t)}(\mathbf{P})^{(t)}y \end{bmatrix}.$$
8. Take the derivative of the log-likelihood function with respect to σ_g^2 and σ_e^2:
$$U = -\frac{1}{2}\begin{bmatrix} \text{tr}\left((\mathbf{P})^{(t)}\mathbf{K} - y^T(\mathbf{P})^{(t)}\mathbf{K}(\mathbf{P})^{(t)}y\right) \\ \text{tr}\left((\mathbf{P})^{(t)} - y^T(\mathbf{P})^{(t)}(\mathbf{P})^{(t)}y\right) \end{bmatrix}.$$
9. Update parameters:
$$\begin{bmatrix} (\sigma_g^2)^{(t+1)} \\ (\sigma_e^2)^{(t+1)} \end{bmatrix} = \begin{bmatrix} (\sigma_g^2)^{(t)} \\ (\sigma_e^2)^{(t)} \end{bmatrix} + \mathbf{Q} + U$$
Go back to step 1 of the AI algorithm with $(t+1)$ parameters until the stopping rule is met.

S2.3 Computational Efficiency

S2.3.1 Efficient Computation of LD scores from Non-scaled Kinship Matrix

The correlation matrix is represented as $\hat{\mathbf{R}} = \frac{\mathbf{X}^T\mathbf{X}}{n}$. Recall that the LD Score of each jth SNP is the sum of all correlations to other k SNPs around it and is computed by squaring each element $\hat{r}_{jk}$ in $\hat{\mathbf{R}}$ and summing across rows:
$$\hat{\ell}_j = \sum_{k=1}^{m} \hat{r}_{jk}^2$$
This is equivalent to computing the diagonal of $\hat{\mathbf{R}}^2 = \frac{1}{n^2}\mathbf{X}^T\mathbf{X}\mathbf{X}^T\mathbf{X}$:
$$\text{diag}(\hat{\mathbf{R}}^2)_{jj} = \sum_{k=1}^{m} \hat{r}_{jk}\hat{r}_{kj} = \sum_{k=1}^{m} \hat{r}_{jk}\hat{r}_{jk} = \sum_{k=1}^{m} (\hat{r}_{jk})^2 = \hat{\ell}_j.$$
Since only the diagonal of $\hat{\mathbf{R}}^2$ is needed, the operation is simplified using the Kinship Matrix: $\mathbf{K} = \frac{\mathbf{X}\mathbf{X}^T}{m}$ and the jth column of $\mathbf{X}$ instead of computing the full matrix:
$$\hat{\ell}_j = \frac{m}{n^2}\mathbf{X}_j^T\mathbf{K}\mathbf{X}_j. \tag{S11}$$

S2.3.2 Efficient Computation of $\hat{\mu}_2$ and $\hat{\mu}_3$

As shown in [10] and in our work here, $\hat{\mu}_2$ and $\hat{\mu}_3$ from Section 2.3.3 require the computation of $\text{tr}(\hat{\mathbf{R}}^2)$ and $\text{tr}(\hat{\mathbf{R}}^3)$ respectively where $\hat{\mathbf{R}} = \frac{\mathbf{X}^T\mathbf{X}}{n}$ is the $m \times m$ correlation matrix and $\mathbf{X}$ is the usual $n \times m$ genotype matrix.

We start with a general case to compute the trace of $\hat{\mathbf{R}}^k$:
$$\text{tr}(\hat{\mathbf{R}}^k) = \text{tr}\left(\left(\frac{\mathbf{X}^T\mathbf{X}}{n}\right)^k\right) = \frac{1}{n^k}\text{tr}\left(\mathbf{X}^T\mathbf{X}...\mathbf{X}^T\mathbf{X}\right). \tag{S12}$$

bioRxiv preprint doi: https://doi.org/10.64898/2026.05.13.724972; this version posted May 16, 2026. The copyright holder for this preprint (which was not certified by peer review) is the author/funder, who has granted bioRxiv a license to display the preprint in perpetuity. It is made available under a CC-BY 4.0 International license.

We use the cyclic property of traces, where for any conformable matrices $\mathbf{A}$ and $\mathbf{B}$, $\text{tr}(\mathbf{AB}) = \text{tr}(\mathbf{BA})$ (see Equation 14 in [8]). Moving $\mathbf{X}$ at the end to the front, Equation (S12) becomes:

$$\text{tr}(\hat{\mathbf{R}}^k) = \frac{1}{n^k}\text{tr}(\mathbf{X}\mathbf{X}^T ... \mathbf{X}\mathbf{X}^T). \tag{S13}$$

The kinship matrix is defined as $\mathbf{K} = \frac{\mathbf{X}\mathbf{X}^T}{m}$ and is $n \times n$. It can be seen from this that $\mathbf{X}\mathbf{X}^T = m\mathbf{K}$. Then, (S13) becomes:

$$\text{tr}(\hat{\mathbf{R}}^k) = \frac{1}{n^k}\text{tr}(m^k\mathbf{K}^k) = \frac{m^k}{n^k}\text{tr}(\mathbf{K}^k). \tag{S14}$$

Computation of $\mathbf{K}^k$ is much more manageable as $n << m$. To compute $\hat{\mu}_2$ and $\hat{\mu}_3$, k is set to 2 and 3 respectively.

The following table summarizes the average computational advantage of utilizing this trick over a direct computation across 100 simulations of $\mathbf{X}$ with AR($\rho = 0.2$) correlation structure.

Estimator	Computation Time Direct $\hat{\mu}_2$ (s)	Computation Time Kinship $\hat{\mu}_2$ (s)	Absolute Difference
$\hat{\mu}_2$	0.1888	0.0029	5.4401e-16
$\hat{\mu}_3$	0.3140	0.0051	3.0531e-15

Table 2: Comparison of kinship method and direct computation for computing $\hat{\mu}_2$ and $\hat{\mu}_3$. The kinship method offers a near 64 fold improvement for $\hat{\mu}_2$ and a near 61 fold improvement for $\hat{\mu}_3$ compared to the direct calculation. The absolute differences between the estimates are extremely small.

S2.3.3 Efficient Computation of Realistic X

Given a real genotype $n \times m$ matrix $\mathbf{X}^*$ that acts as a reference panel, we want a desired $\mathbf{X}$ simulated with similar LD structure that has dimensions $k \times m$. Here, n is the sample size of the real genotype matrix $\mathbf{X}^*$ and k is the sample size of the desired simulated genotype matrix. Both $\mathbf{X}$ and $\mathbf{X}^*$ must share the same m SNPs.

In twas_sim [11], $\mathbf{X}^*$ undergoes pre-processing to ensure that $\frac{1}{n}\sum_{i=1}^n X_{ij}^* = 0$ and $\frac{1}{n}\sum_{i=1}^n \left(X_{ij}^*\right)^2 = 1$ for all j. Then, $\mathbf{X}$ is generated from a lower-triangular of $(\mathbf{X}^*)^T\mathbf{X}^*$, which is denoted by $\mathbf{L}$, and a matrix $\mathbf{Z}$ of dimensions $k \times m$ with iid standard normal entries independent of $\mathbf{X}^*$:

$$\mathbf{X} = \frac{1}{\sqrt{m}}(\mathbf{L}\mathbf{Z}^T)^T$$

This induces correlation between columns following the LD structure in $\mathbf{L}$. It is easy to see that in expectation $\mathbf{X}^T\mathbf{X}$ is equal to $(\mathbf{X}^*)^T\mathbf{X}^*$:

$$\text{E}\left[\mathbf{X}^T\mathbf{X}|\mathbf{X}^*\right] = \frac{1}{m}\text{E}\left[(\mathbf{L}\mathbf{Z}^T)(\mathbf{L}\mathbf{Z}^T)^T|\mathbf{X}^*\right] = \frac{1}{m}\mathbf{L}\text{E}\left[\mathbf{Z}^T\mathbf{Z}\right]\mathbf{L}^T = \mathbf{L}\mathbf{L}^T = (\mathbf{X}^*)^T\mathbf{X}^*.$$

A well-known requirement of using the Cholesky Decomposition method is that the matrix must be positive definite. To ensure that $(\mathbf{X}^*)^T\mathbf{X}^*$ matches such a condition, a diagonal matrix of some arbitrary coefficient ld_{ridge} is added as a correction matrix [5] and the matrix $(\mathbf{X}^*)^T\mathbf{X}^*$ is standardized by $1 + \text{ld}_{\text{ridge}}$ to ensure that the diagonal of the new corrected LD matrix is 1. If ld_{ridge} is small, the off diagonals should be offset by a near-negligible factor. To do Cholesky Decomposition in twas_sim, ld_{ridge} is set to 0.1, and this value can significantly offset the off-diagonal values. Further, $\mathbf{X}$ is standardized such that $\frac{1}{n}\sum_{i=1}^n X_{ij} = 0$ and $\frac{1}{n}\sum_{i=1}^n X_{ij}^2 = 1$ for all j.

We show that the same objective can be met without doing Cholesky Decomposition and eliminating this correction cost. Specifically, let $\mathbf{Z}$ be a $k \times n$ matrix with iid standard normal entries independent of $\mathbf{X}^*$, and define

$$\mathbf{X} = \frac{1}{\sqrt{n}}\mathbf{Z}\mathbf{X}^*.$$

bioRxiv preprint doi: https://doi.org/10.64898/2026.05.13.724972; this version posted May 16, 2026. The copyright holder for this preprint (which was not certified by peer review) is the author/funder, who has granted bioRxiv a license to display the preprint in perpetuity. It is made available under a CC-BY 4.0 International license.

Repeating a similar calculation as above we have

$$\mathrm{E}\left[\mathbf{X}^T\mathbf{X}|\mathbf{X}^*\right] = \frac{1}{n}\mathrm{E}\left[(\mathbf{X}^*\mathbf{Z}^T)(\mathbf{X}^*\mathbf{Z}^T)^T|\mathbf{X}^*\right] = \frac{1}{n}\mathbf{X}^*\mathrm{E}\left[\mathbf{Z}^T\mathbf{Z}\right]\left(\mathbf{X}^*\right)^T = \left(\mathbf{X}^*\right)^T\mathbf{X}^*.$$

Then, the matrix $\mathbf{X}$ undergoes the same standardization as that from twas_sim to ensure $\frac{1}{n}\sum_{i=1}^n X_{ij} = 0$ and $\frac{1}{n}\sum_{i=1}^n X_{ij}^2 = 1$ for all j.

S2.4 Modifications to GWASH to match LDSC in Real Data

The goal of this section is to clarify implementation details to enable a direct comparison between LDSC and GWASH. This direct comparison is possible because GWASH should be close to LDSC with fixed intercept as discussed in [10]. These implementation details are the following:

1. To mirror the analysis done in the LDSC package, in the LDSC calculation we set $m = \sum_{j=1}^{m_{\mathrm{ref}}} I(\mathrm{MAF}_j > 0.05)$, where m_{ref} is the number of SNPs in the reference panel and MAF_j is the minor allele frequency for the jth SNP. Note that this m refers to the number of SNPs used in the calculation of LD scores, and is therefore different from the number of SNPs that overlap between the reference panel and dataset. This is only done in the real data analysis as we assume in simulations that all SNPs in the reference panel match those in the simulated dataset and meet the $\mathrm{MAF}_j > 0.05$ criteria.
2. We multiply $\hat{\mu}_2$ (see Equation (10)) and $\hat{\mu}_3$ (see Equation (13)) by a factor of $\frac{n-1}{n-2}$. We denote these quantities as $\tilde{\mu}_2$ and $\tilde{\mu}_3$ which consider the bias correction discussed in [3]. This is done in both real data and simulations.
3. In $\tilde{\mu}_2$ and $\tilde{\mu}_3$, m is used instead of $m-1$ because of how $\tilde{\ell}_j$ is computed. This is only done in the real data analysis.
4. The quantities $\tilde{\mu}_2$ and $\tilde{\mu}_3$ are computed with a 1 Centimorgan window similarly to how this window is used in LDSC. We then compute $\tilde{\mu}_2$ and $\tilde{\mu}_3$ by chromosome as specified in [10]. This is only done in the real data analysis.

We discuss each point in a respective section below.

S2.4.1 Rationale of using $m = \sum_{j=1}^{m_{\mathrm{ref}}} I(\mathrm{MAF}_j > 0.05)$ in LDSC

In the LDSC software package [3], $\tilde{\ell}_j^*$ from a reference panel must be prepared. Each $\tilde{\ell}_j^*$ is computed with all SNPs found in the reference panel which may not include SNPs found in the dataset. Then, m is set to $\sum_{j=1}^{m_{\mathrm{ref}}} I(\mathrm{MAF}_j > 0.05)$ from the reference panel to account for the "missing" SNPs not found in the dataset that contribute LD in $\tilde{\ell}_j^*$. The value of m in the LDSC calculation refers to the number of SNPs used in the calculation of LD scores, not the number of SNPs that overlap between the reference panel and dataset. If m was to be set to the number of SNPs that overlap the reference panel and dataset, this might not accurately represent $\tilde{\ell}_j^*$ (as this considers the mismatched SNPs). It could also inflate $\hat{h}^2$ because the dominator is smaller since $m_{\mathrm{ref}} > m$. In simulations, we assume the ideal case that all SNPs in the reference panel are found in the dataset and that all SNPs have $\mathrm{MAF}_j > 0.05$.

S2.4.2 Computation of $\tilde{\mu}_2$ and $\tilde{\mu}_3$

We make the deliberate choice of adjusting the GWASH equations in the implementation by a factor of $\frac{n-1}{n-2}$. This is because the LD scores $\tilde{\ell}_j$ (as defined in Equation (3)) have a slightly different bias correction term than that presented in [10]. At low n, such as with the 1000 Genomes Phase 1 reference panel with an $n = 378$, this discrepancy is notable. We show this by examining the relative difference across different n sample sizes in simulation.

bioRxiv preprint doi: https://doi.org/10.64898/2026.05.13.724972; this version posted May 16, 2026. The copyright holder for this preprint (which was not certified by peer review) is the author/funder, who has granted bioRxiv a license to display the preprint in perpetuity. It is made available under a CC-BY 4.0 International license.

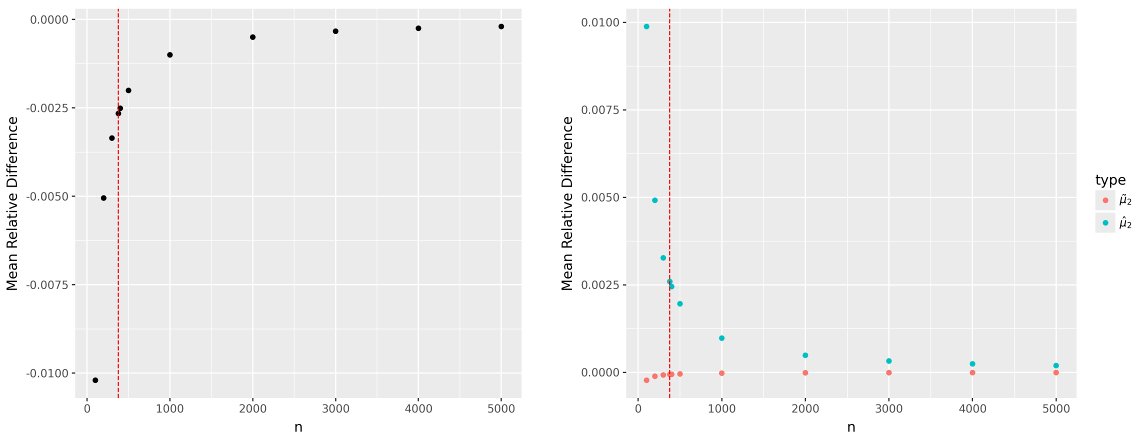

Figure S2: Mean relative difference between $\tilde{\mu}_2$ and $\hat{\mu}_2$ across 100 realistic LD simulations of $m = 10000$ across varying n (left panel) and mean relative difference between these quantities and the average of $\tilde{\boldsymbol{\ell}}$. As n increases, the difference of the mean of $\tilde{\ell}_j$ with $\hat{\mu}_2$ of the 2019 equation decays exponentially. From the right panel, we can see that the mean relative difference of both $\tilde{\mu}_2$ and $\hat{\mu}_2$ against the average of $\hat{\boldsymbol{\ell}}$ decreases towards 0 as n increases. Note that the y-axis for both panels is not shared. The red dotted line in both panels represents the sample size of the actual 1000G Phase 1 reference panel.

Recall that $\hat{\mu}_2$ is written as the average of $\hat{\ell}_j$'s and we define $\tilde{\mu}_2$ as the average of $\tilde{\ell}_j$'s, i.e., $\tilde{\mu}_2 := \frac{1}{m}\sum_{j=1}^m \tilde{\ell}_j$. In the left panel of Figure S2, the relative difference between $\tilde{\mu}_2$ and $\hat{\mu}_2$ is computed as $1 - \frac{\tilde{\mu}_2}{\hat{\mu}_2}$. In the right panel, the relative differences with respect to $\frac{1}{m}\sum_{j=1}^m \tilde{\ell}_j$ are computed as

$$1 - \frac{\tilde{\mu}_2}{\frac{1}{m}\sum_{j=1}^m \tilde{\ell}_j}, \qquad \text{and} \qquad 1 - \frac{\hat{\mu}_2}{\frac{1}{m}\sum_{j=1}^m \tilde{\ell}_j}.$$

The difference between $\hat{\mu}_2$ from the implementation in [10] and the mean $\tilde{\ell}_j$ is quite substantial at $n = 378$. If $\hat{\mu}_2$ is not adjusted, LDSC and GWASH with real data are not comparable.

Below, we show that $\frac{n-1}{n-2}\hat{\mu}_2 \approx \tilde{\mu}_2$. We use the result from the middle part of Equation (S8) in Section S1.2.2:

$$\begin{aligned}\tilde{\ell}_j &= \sum_{k=1}^m \hat{r}_{jk}^2 - \left(\frac{m}{n-2} - \sum_{k=1}^m \frac{\hat{r}_{jk}^2}{n-2}\right)\\ &= \hat{\ell}_j + \frac{1}{n-2}\hat{\ell}_j - \frac{m}{n-2}\\ &= \frac{n-1}{n-2}\hat{\ell}_j - \frac{m}{n-2}.\end{aligned}$$

Now,

$$\begin{aligned}\tilde{\mu}_2 &= \frac{1}{m}\sum_{j=1}^m \tilde{\ell}_j = \frac{1}{m}\sum_{j=1}^m \left(\frac{n-1}{n-2}\hat{\ell}_j - \frac{m}{n-2}\right)\\ &= \frac{1}{m}\left[\frac{n-1}{n-2}\sum_{j=1}^m \hat{\ell}_j - \frac{m^2}{n-2}\right].\end{aligned} \tag{S15}$$

We have that $\mathrm{tr}(\hat{\mathbf{R}}^2) = \sum_{j=1}^m \hat{\ell}_j$ because

$$\hat{\mathbf{R}}_{jj}^2 = \sum_{k=1}^m \hat{r}_{jk}\hat{r}_{kj} = \sum_{k=1}^m \hat{r}_{jk}^2 = \hat{\ell}_j,$$

bioRxiv preprint doi: https://doi.org/10.64898/2026.05.13.724972; this version posted May 16, 2026. The copyright holder for this preprint (which was not certified by peer review) is the author/funder, who has granted bioRxiv a license to display the preprint in perpetuity. It is made available under a CC-BY 4.0 International license.

and therefore $\text{tr}(\hat{\mathbf{R}}^2) = \sum_{j=1}^{m} \hat{\mathbf{R}}_{jj}^2 = \sum_{j=1}^{m} \hat{\ell}_j$. Equation (S15) becomes

$$\tilde{\mu}_2 = \frac{n-1}{n-2}\frac{1}{m}\text{tr}(\hat{\mathbf{R}}^2) - \frac{m}{n-2} \tag{S16}$$

and one can see that Equation (S16) is approximately Equation (10) scaled by a factor of $\frac{n-1}{n-2}$:

$$\begin{aligned}\tilde{\mu}_2 &= \left(\frac{n-1}{n-2}\right)\left(\frac{1}{m}\text{tr}(\hat{\mathbf{R}}^2) - \frac{m}{n-1}\right)\\ &\approx \left(\frac{n-1}{n-2}\right)\left(\frac{1}{m}\text{tr}(\hat{\mathbf{R}}^2) - \frac{m-1}{n-1}\right)\\ &= \frac{n-1}{n-2}\hat{\mu}_2.\end{aligned}$$

The $\frac{n-1}{n-2}$ term is also multiplied to $\hat{\mu}_3$ in our implementation:

$$\tilde{\mu}_3 = \frac{n-1}{n-2}\hat{\mu}_3 = \frac{1}{m}\frac{n-1}{n-2}\text{tr}(\hat{\mathbf{R}}^3) - \frac{3m}{n-2}\hat{\mu}_2 - \frac{m^2}{(n-1)(n-2)}.$$

Notice that m is used instead of $m-1$ as written in Equation (10) in the main text. This comes from the usage of $\tilde{\ell}_j$. We address this in the next section.

S2.4.3 Use of m instead of $m-1$ in $\tilde{\ell}_j$, $\tilde{\mu}_2$, and $\tilde{\mu}_3$.

We also note that the equations for $\tilde{\mu}_2$ and $\tilde{\mu}_3$ presented in this implementation and [10] have $m-1$ instead of m as shown in $\tilde{\mu}_2$ and $\tilde{\mu}_3$. We justify using m for the real dataset analysis to directly compare GWASH and LDSC since we use the released precomputed LD scores while we use $m-1$ as written in the main text equations for the simulations.

If m is replaced by $m-1$ in the original equation for $\tilde{\ell}_j$ (S2.4.2), then the computation of $\tilde{\mu}_2$ from individual-level data above will equal the average of all $\tilde{\ell}_j$s within a numerical accuracy of $\sim 10^{-15}$. We show this empirically when varying n and m of 1000 simulations. We assume that the reference panel and dataset have the same sample size and number of SNPs.

n	m	use $m-1$	$\tilde{\mu}_2$ from $\tilde{\ell}_j$	$\tilde{\mu}_2$ from reference $\mathbf{X}$	absolute difference
50	100	True	1.9211e+01 (5.2815e+00)	1.9211e+01 (5.2815e+00)	2.9061e-15 (2.8765e-15)
50	100	False	1.9190e+01 (5.2815e+00)	1.9211e+01 (5.2815e+00)	2.0833e-02 (4.1715e-15)
500	1000	True	1.9480e+01 (5.8048e-01)	1.9480e+01 (5.8048e-01)	2.5224e-15 (2.4777e-15)
500	1000	False	1.9478e+01 (5.8048e-01)	1.9480e+01 (5.8048e-01)	2.0080e-03 (3.1034e-15)
5000	10000	True	1.9510e+01 (5.8228e-02)	1.9510e+01 (5.8228e-02)	2.4798e-15 (2.2782e-15)
5000	10000	False	1.9510e+01 (5.8228e-02)	1.9510e+01 (5.8228e-02)	2.0008e-04 (3.2764e-15)

Table 3: Comparison of $\tilde{\mu}_2$ computed from taking the mean of $\tilde{\ell}_j$s against $\tilde{\mu}_2$ computed directly from $\mathbf{X}$ across different n and m using m as done in [3] or $m-1$ as done in [10]. This analysis was conducted over 1000 simulations.

Across 1000 simulations, the average absolute difference of $\tilde{\mu}_2$ decreases by a factor of 10. This is because $m-1 \approx m$ as m increases. We emphasize that $m-1$ in the equations of the main text is replaced with m for the real data analysis but is as written for simulations.

S2.4.4 Computation of $\tilde{\mu}_2$ and $\tilde{\mu}_3$ by chromosome on 1000 Genomes Data with 1 Centimorgan Window

To directly compare GWASH and LDSC on real datasets, the quantities that are used in each estimator must be preprocessed in the same way. We follow the strategy outlined in [10] to compute the LD contributions

bioRxiv preprint doi: https://doi.org/10.64898/2026.05.13.724972; this version posted May 16, 2026. The copyright holder for this preprint (which was not certified by peer review) is the author/funder, who has granted bioRxiv a license to display the preprint in perpetuity. It is made available under a CC-BY 4.0 International license.

within each chromosome and then combine the results across them. As also done in LDSC, for each jth SNP, we compute 1 Centimorgan SNP correlation blocks, apply the bias correction as discussed previously in Section S2.4.2, and create $\hat{\mathbf{R}}_k$ for each kth chromosome. We then compute the contribution of each jth SNP to $(\hat{\mu}_2)_k$ for each kth chromosome:

$$((\tilde{\mu}_2)_k)_j = \frac{n-1}{n-2}\left(\text{diag}(\hat{\mathbf{R}}_k^2)_j - \frac{1}{n-1}\right)$$
$$(\tilde{\mu}_2)_k = \frac{1}{m_k}\sum_{j=1}^{m_k}((\tilde{\mu}_2)_k)_j$$

where $\text{diag}(\hat{\mathbf{R}}_k^2)_j$ is the jth entry of the diagonal of $\hat{\mathbf{R}}_k^2$, the covariance matrix of SNPs in chromosome k. We also do a similar operation for $(\tilde{\mu}_3)_k$:

$$((\tilde{\mu}_3)_k)_j = \frac{n-1}{n-2}\left(\text{diag}(\hat{\mathbf{R}}_k^3)_j - 3\frac{1}{n-1} - \frac{m_k}{(n-1)^2}\right)$$
$$(\tilde{\mu}_3)_k = \frac{1}{m_k}\sum_{j=1}^{m_k}((\tilde{\mu}_3)_k)_j$$

where m_k is the number of SNPs on the kth chromosome. We note that m_k is used here instead of $m_k - 1$ as written in Equation (10) and Equation (13) to facilitate direct comparisons to LDSC. We store $((\tilde{\mu}_2)_k)_j$ and $((\tilde{\mu}_3)_k)_j$ in separate tables that can be readily loaded. We then compute $\tilde{\mu}_2$ by computing the weighted average of all $(\tilde{\mu}_2)_k$s across the k chromosomes:

$$\tilde{\mu}_2 = \sum_{k=1}^{22}(\tilde{\mu}_2)_k * \frac{m_k}{m}$$

and $\tilde{\mu}_3$ as a weighted average of $(\tilde{\mu}_3)_k$s:

$$\tilde{\mu}_3 = \sum_{k=1}^{22}(\tilde{\mu}_3)_k * \frac{m_k}{m}.$$

The code to compute all of these quantities described can be found on our GitHub repository (see Section 7).

S2.5 GWASH SE formula breaks at high ρ

The standard error formula for GWASH from [10] works when $\rho < 0.8$. At higher ρ values, we see that the GWASH standard error formula underestimates. The true GWASH se is calculated from the exact AR1 ρ correlation matrix and $h_g^2 = 0.2$.

bioRxiv preprint doi: https://doi.org/10.64898/2026.05.13.724972; this version posted May 16, 2026. The copyright holder for this preprint (which was not certified by peer review) is the author/funder, who has granted bioRxiv a license to display the preprint in perpetuity. It is made available under a CC-BY 4.0 International license.

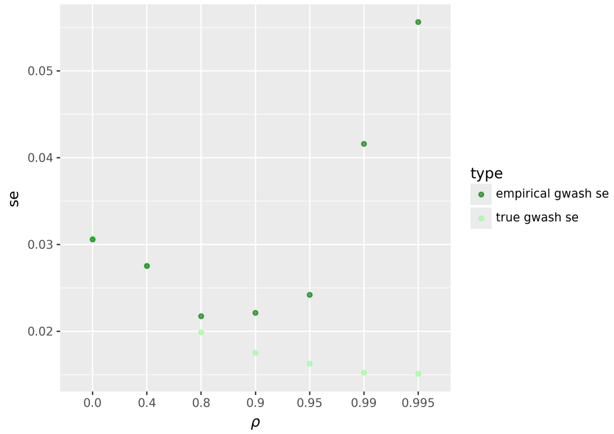

Figure S3: Comparison of empirical GWASH se from simulation against GWASH se computed with the true AR1 correlation matrix and $h_g^2 = 0.2$. The previous study [10] only looked at $\rho \leq 0.8$ where the formula and empirical se mostly aligns. At higher ρ regimes, the formula underestimates the empirical se from simulations.

The observed AR1 simulation standard errors are underestimated because the default ρ for the AR1 simulations is 0.995. Although the Realistic Simulations do not have AR1 correlation structure, we see the same underestimation effect in the estimated standard errors.

S3 $\text{prop}_{\text{causal}}$ Simulations with Reference Panel

We observed that changing $\text{prop}_{\text{causal}}$ has no effect on heritability estimation in both AR1 and realistic LD simulations. These figures are shown here.

S3.1 AR1 $\text{prop}_{\text{causal}}$ Simulations with reference panel

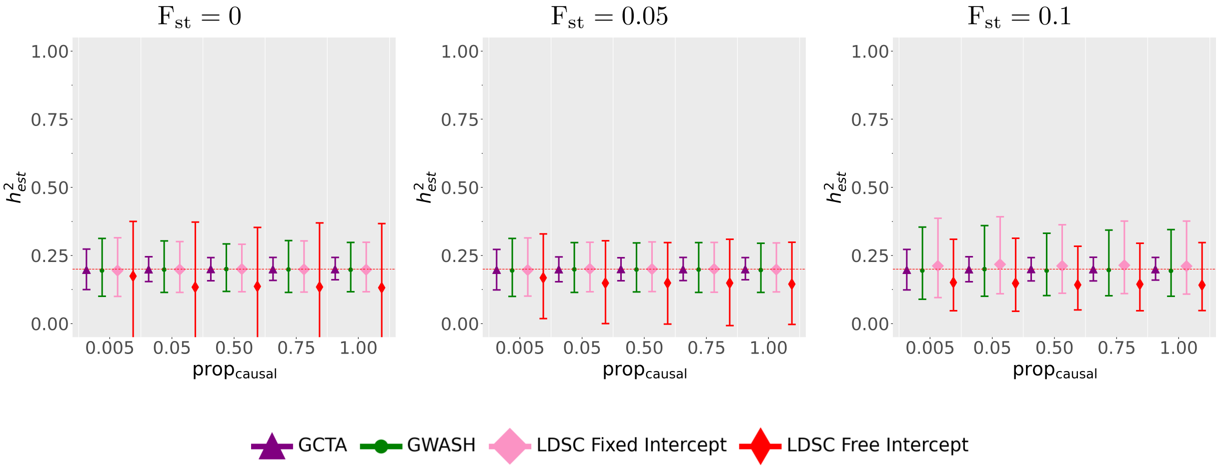

Figure S4: Heritability estimates on simulated data across 1000 simulations where a parameter of interest is gradually increased from $F_{st} = 0$ (left panel) to $F_{st} = 0.05$ (middle panel) and $F_{st} = 0.1$ (right panel). The setting is the same as in Figure 1.

bioRxiv preprint doi: https://doi.org/10.64898/2026.05.13.724972; this version posted May 16, 2026. The copyright holder for this preprint (which was not certified by peer review) is the author/funder, who has granted bioRxiv a license to display the preprint in perpetuity. It is made available under a CC-BY 4.0 International license.

S3.2 Realistic $\text{prop}_{\text{causal}}$ Simulations with reference panel

Like with the AR1 simulations, we observe that $\text{prop}_{\text{causal}}$ has no effect on heritability estimation.

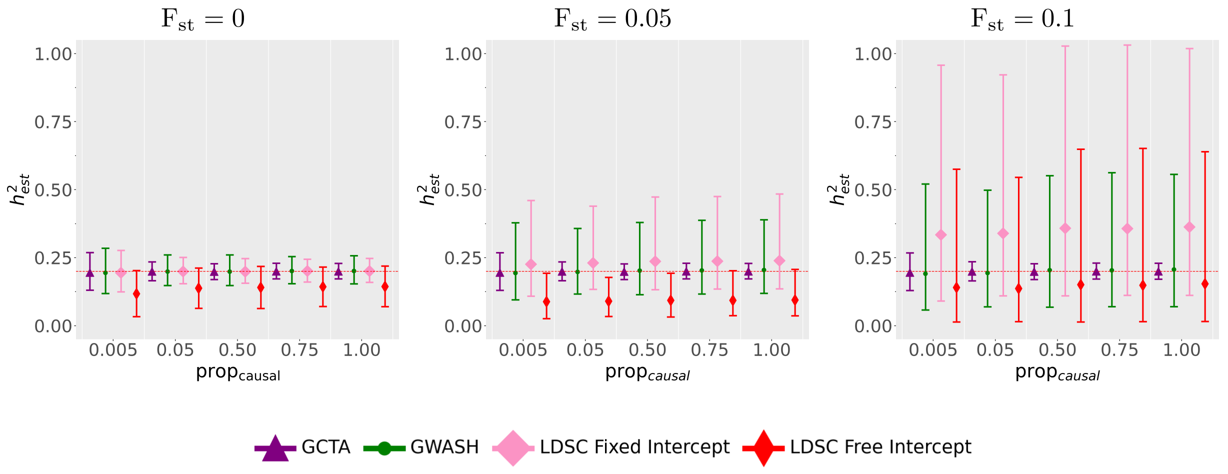

Figure S5: Heritability estimates on simulated data across 1000 simulations where a parameter of interest is gradually increased from $F_{st} = 0$ (left panel) to $F_{st} = 0.05$ (middle panel) and $F_{st} = 0.1$ (right panel). The setting is the same as in Figure 2.

S3.3 AR1 $\text{prop}_{\text{causal}}$ Standard Error Estimation

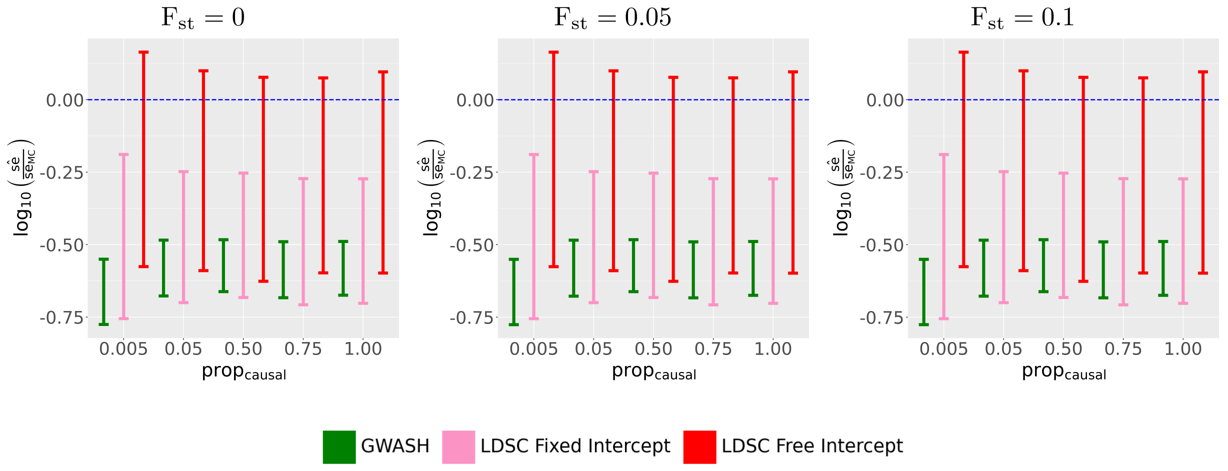

Figure S6: Heritability estimates on simulated data across 1000 simulations where a parameter of interest is gradually increased from $F_{st} = 0$ (left panel) to $F_{st} = 0.05$ (middle panel) and $F_{st} = 0.1$ (right panel). The setting is the same as in Figure 3.

bioRxiv preprint doi: https://doi.org/10.64898/2026.05.13.724972; this version posted May 16, 2026. The copyright holder for this preprint (which was not certified by peer review) is the author/funder, who has granted bioRxiv a license to display the preprint in perpetuity. It is made available under a CC-BY 4.0 International license.

S3.4 Realistic $\text{prop}_{\text{causal}}$ Standard Error Estimation

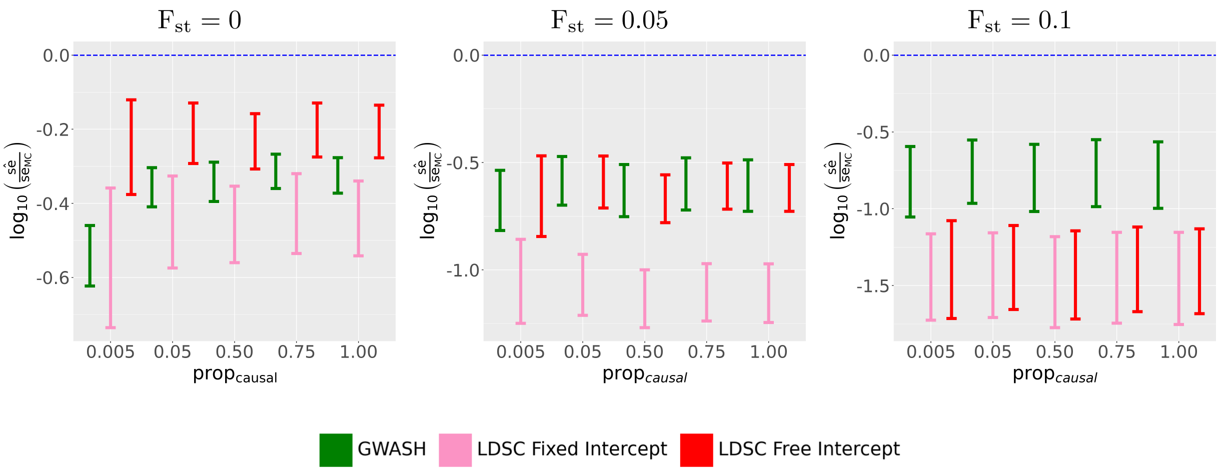

Figure S7: Heritability estimates on simulated data across 1000 simulations where a parameter of interest is gradually increased from $F_{st} = 0$ (left panel) to $F_{st} = 0.05$ (middle panel) and $F_{st} = 0.1$ (right panel). The setting is the same as in Figure 4.

S3.5 Impact of standard error estimates on Z-Scores in AR1 Simulations when changing $\text{prop}_{\text{causal}}$

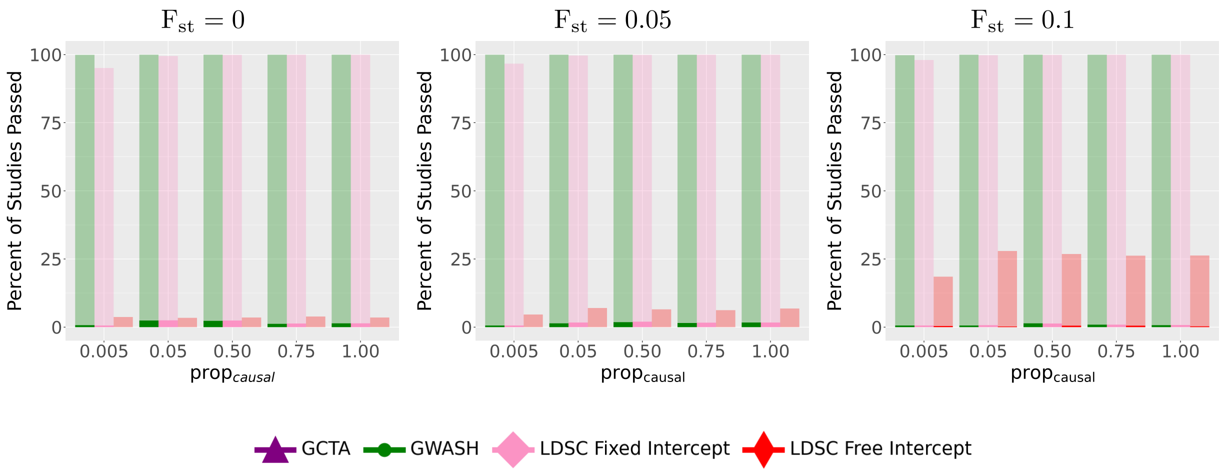

Figure S8: Heritability estimates on simulated data across 1000 simulations where a parameter of interest is gradually increased from $F_{st} = 0$ (left panel) to $F_{st} = 0.05$ (middle panel) and $F_{st} = 0.1$ (right panel). The setting is the same to that in Figure 1.

bioRxiv preprint doi: https://doi.org/10.64898/2026.05.13.724972; this version posted May 16, 2026. The copyright holder for this preprint (which was not certified by peer review) is the author/funder, who has granted bioRxiv a license to display the preprint in perpetuity. It is made available under a CC-BY 4.0 International license.

S3.6 Impact of standard error estimates on Z-Scores in realistic Simulations when changing $\text{prop}_{\text{causal}}$

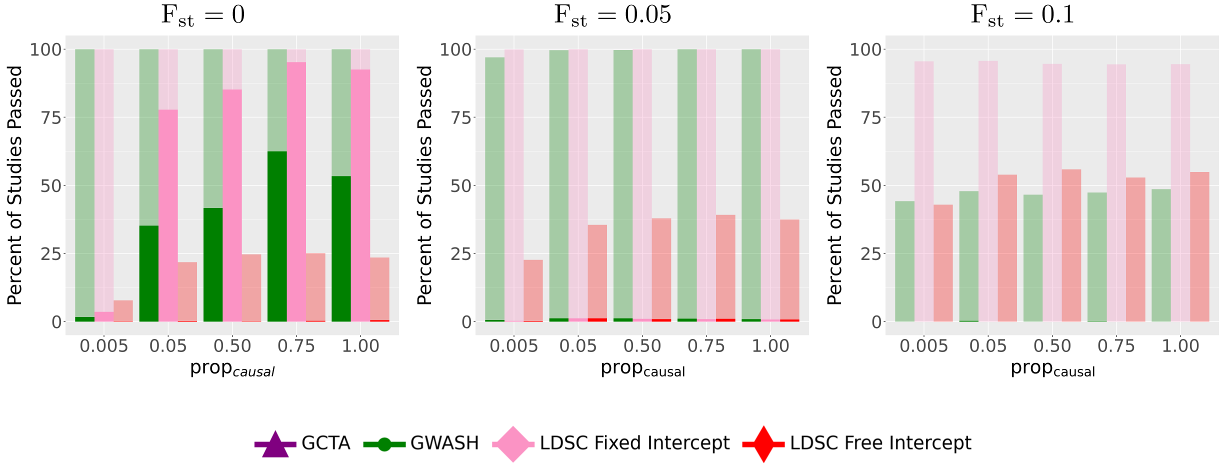

Figure S9: Heritability estimates on simulated data across 1000 simulations where a parameter of interest is gradually increased from $F_{st} = 0$ (left panel) to $F_{st} = 0.05$ (middle panel) and $F_{st} = 0.1$ (right panel). The setting is the same to that in Figure 2.

S4 Simulations with Individual-Level Data

S4.1 AR1 Supplementary Simulations with Individual Level Data

These AR1 simulations have the same setting as those in the main text, using the same reference panel as the individual-level data. This is the most optimistic case where the reference panel matches exactly the individual level data.

bioRxiv preprint doi: https://doi.org/10.64898/2026.05.13.724972; this version posted May 16, 2026. The copyright holder for this preprint (which was not certified by peer review) is the author/funder, who has granted bioRxiv a license to display the preprint in perpetuity. It is made available under a CC-BY 4.0 International license.

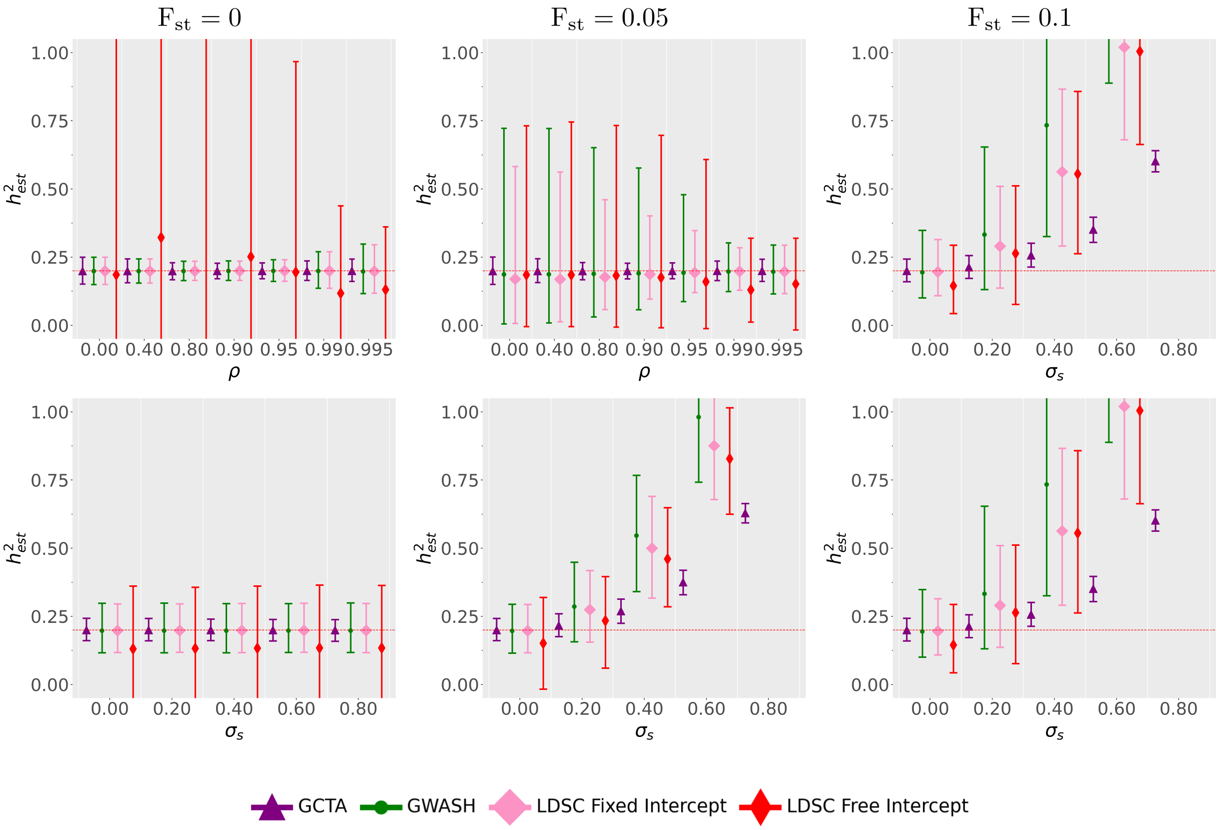

Figure S10: Heritability estimates when changing ρ and σ_s while gradually increasing $\mathrm{F_{st}}$ from $\mathrm{F_{st}} = 0$ (left column) to $\mathrm{F_{st}} = 0.05$ (middle column) and $\mathrm{F_{st}} = 0.1$ (right column). The setting is similar to that in Figure 1 except that the sample data is observed and can thus be used as the reference panel.

S4.2 Realistic Supplementary Simulations with Individual Level Data

These Realistic simulations have the same setting as those in the main text, using the same reference panel as the individual-level data. This is the most optimistic case where the reference panel matches exactly the individual level data.

bioRxiv preprint doi: https://doi.org/10.64898/2026.05.13.724972; this version posted May 16, 2026. The copyright holder for this preprint (which was not certified by peer review) is the author/funder, who has granted bioRxiv a license to display the preprint in perpetuity. It is made available under a CC-BY 4.0 International license.

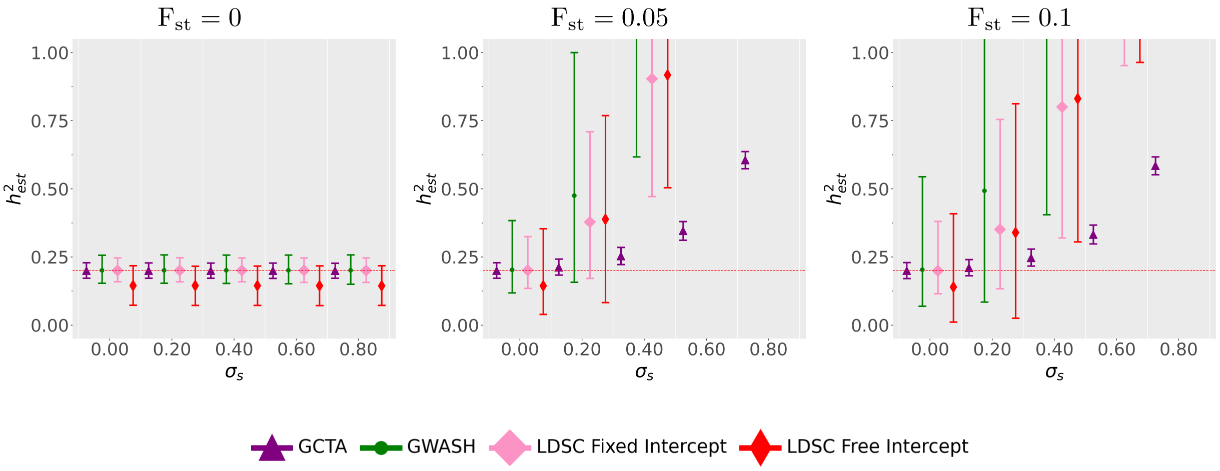

Figure S11: Heritability estimates on simulated data across 1000 simulations where a parameter of interest is gradually increased from $F_{st} = 0$ (left panel) to $F_{st} = 0.05$ (middle panel) and $F_{st} = 0.1$ (right panel). The setting is similar to that in Figure 2 except that the sample data is observed and can thus be used as the reference panel.

S5 Estimation of Standard Error

S5.1 Standard Error AR1 Supplementary Simulations with Individual-Level Data

These Standard Error evaluations correspond to the AR1 Supplementary Figures with full individual-level data.

bioRxiv preprint doi: https://doi.org/10.64898/2026.05.13.724972; this version posted May 16, 2026. The copyright holder for this preprint (which was not certified by peer review) is the author/funder, who has granted bioRxiv a license to display the preprint in perpetuity. It is made available under a CC-BY 4.0 International license.

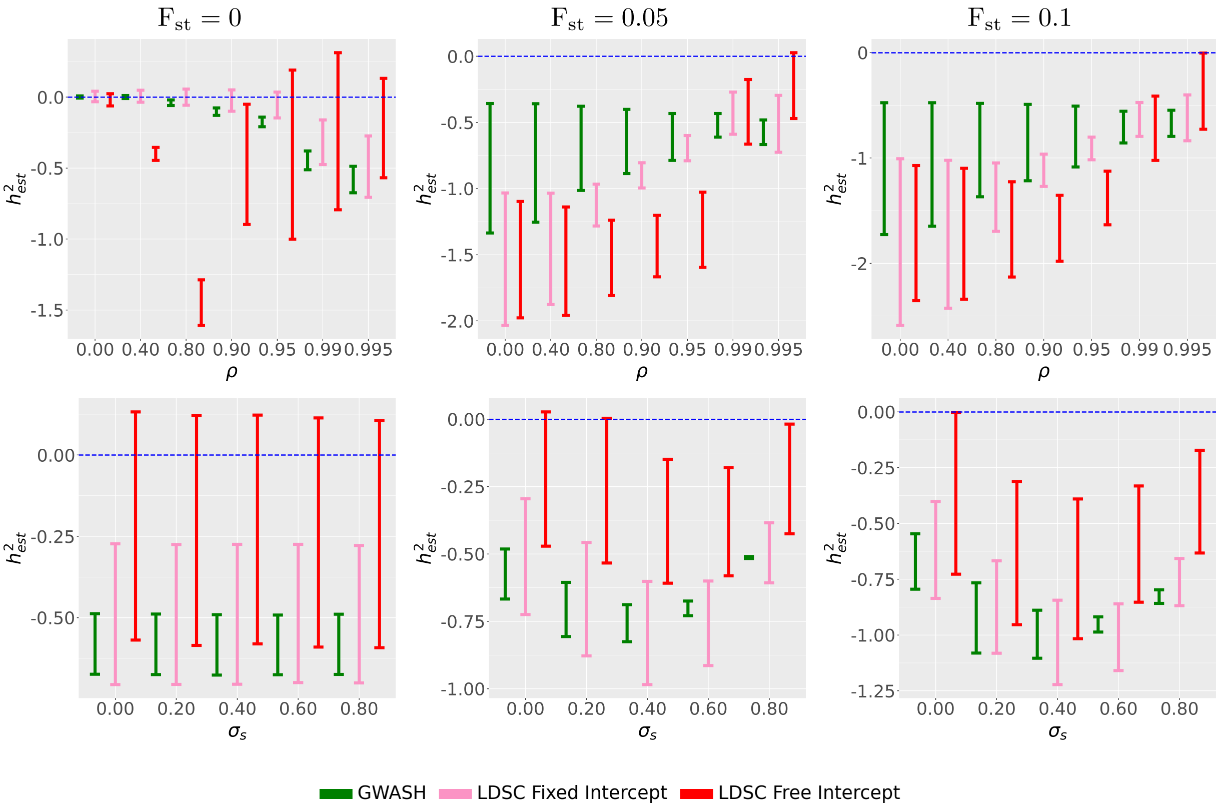

Figure S12: Evaluation of sample standard errors in AR1 simulated data with genetic confounding across 1000 replicates. The setting is the same as in Figure 1 except that the sample data is observed and is used as the reference panel.

S5.2 Standard Error Realistic Supplementary Simulations with Individual-Level Data

These Standard Error evaluations correspond to the Realistic Supplementary Figures with full individual-level data.

bioRxiv preprint doi: https://doi.org/10.64898/2026.05.13.724972; this version posted May 16, 2026. The copyright holder for this preprint (which was not certified by peer review) is the author/funder, who has granted bioRxiv a license to display the preprint in perpetuity. It is made available under a CC-BY 4.0 International license.

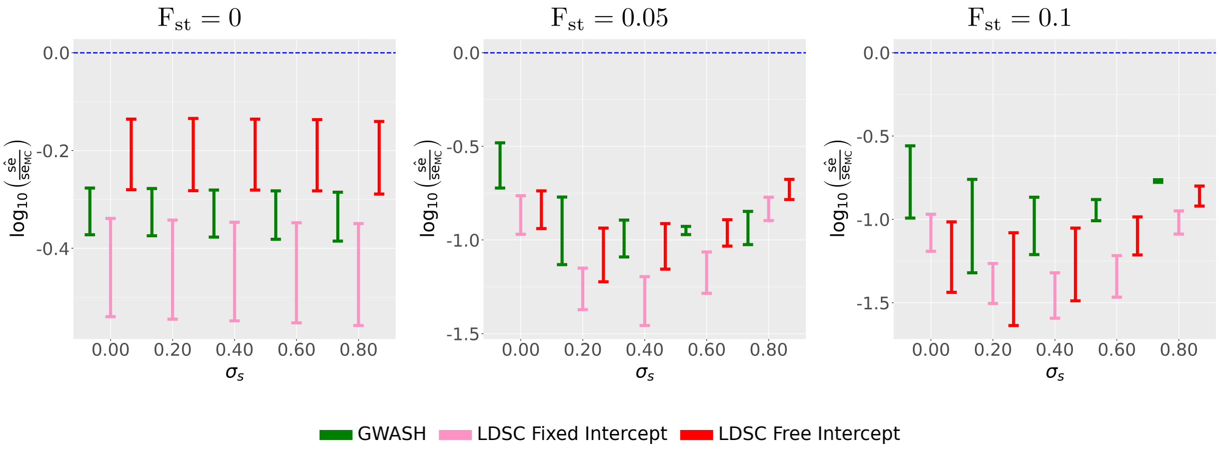

Figure S13: Evaluation of sample standard errors in LD simulated data with genetic confounding across 1000 replicates. The setting is the same as in Figure 4 except that the sample data is observed and is used as the reference panel.

bioRxiv preprint doi: https://doi.org/10.64898/2026.05.13.724972; this version posted May 16, 2026. The copyright holder for this preprint (which was not certified by peer review) is the author/funder, who has granted bioRxiv a license to display the preprint in perpetuity. It is made available under a CC-BY 4.0 International license.

S5.3 Impact of standard error estimates on Z-Scores in Individual-Level AR1 Simulations

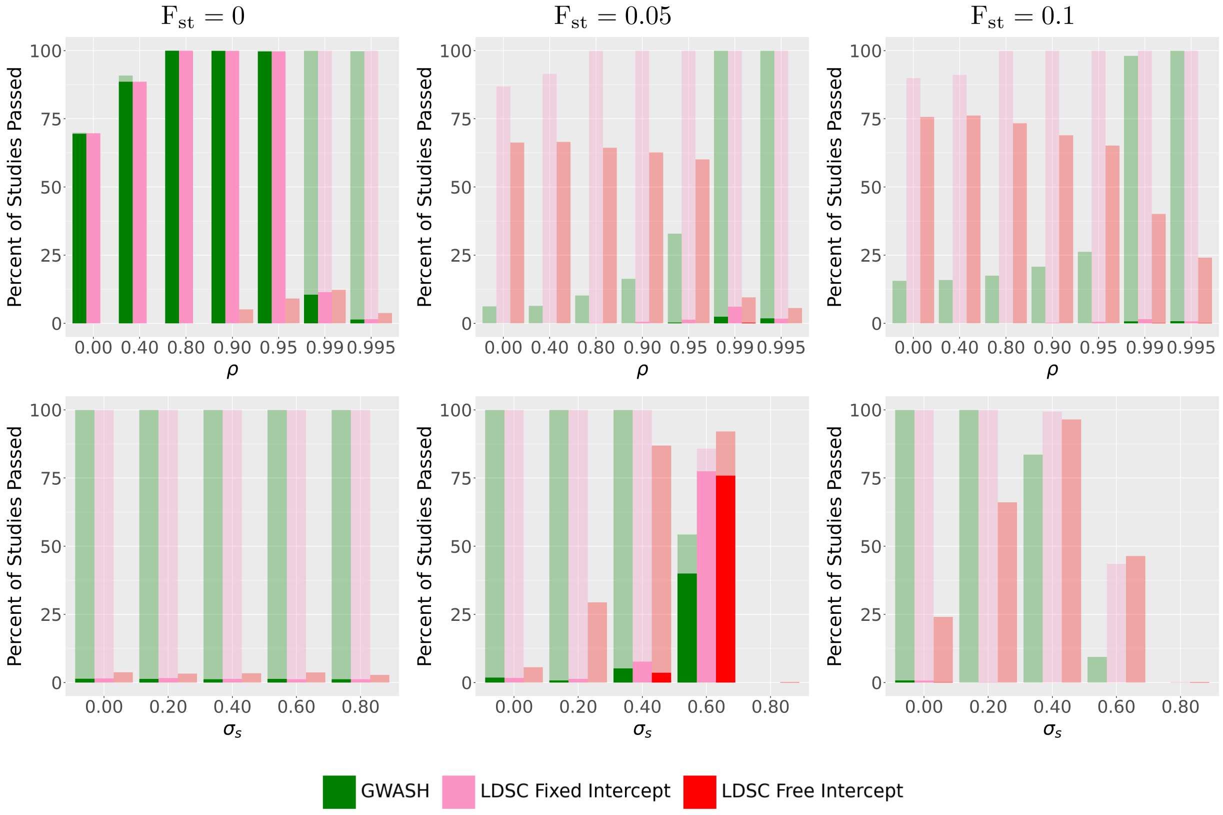

Figure S14: Evaluation of studies passed based on heritability z-scores in AR1 Simulations changing ρ or σ_s after F_{st} is increased. The left column shows no genetic confounding ($F_{st} = 0.0$), the middle column is with moderate genetic confounding ($F_{st} = 0.05$) and the right column is with high genetic confounding ($F_{st} = 0.1$). This figure is the same as Figure 5 except that the sample data is observed and is used as the reference panel.

bioRxiv preprint doi: https://doi.org/10.64898/2026.05.13.724972; this version posted May 16, 2026. The copyright holder for this preprint (which was not certified by peer review) is the author/funder, who has granted bioRxiv a license to display the preprint in perpetuity. It is made available under a CC-BY 4.0 International license.

S5.4 Impact of standard error estimates on Z-Scores in Individual-Level Realistic Simulations

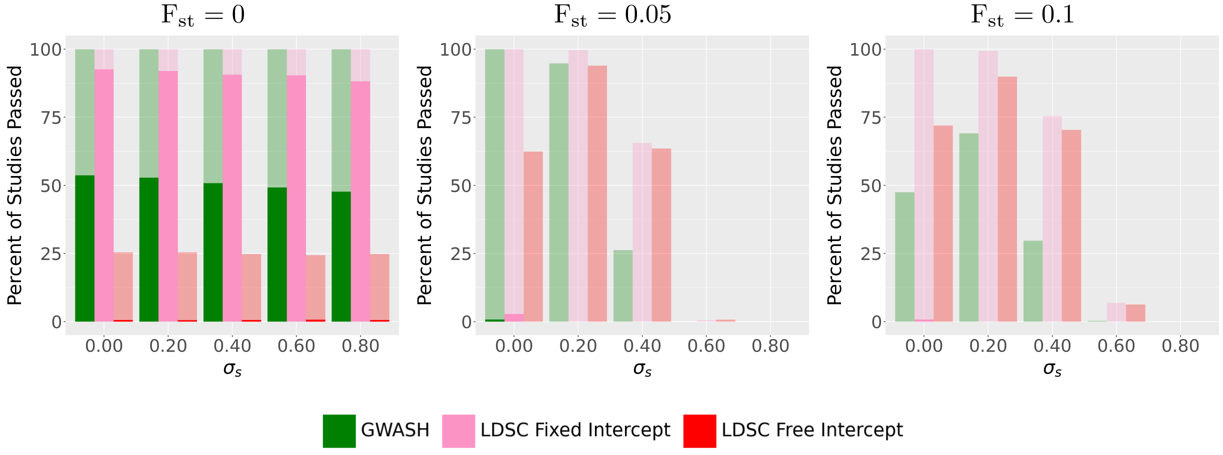

Figure S15: Evaluation of studies passed based on heritability z-scores in Realistic Simulations changing a parameter after F_{st} is increased from $F_{st} = 0$ (left panel) to $F_{st} = 0.05$(middle panel) and $F_{st} = 0.1$ (right panel). This is the same setting as in Figure 6 except that the sample data is observed and is used as the reference panel.

bioRxiv preprint doi: https://doi.org/10.64898/2026.05.13.724972; this version posted May 16, 2026. The copyright holder for this preprint (which was not certified by peer review) is the author/funder, who has granted bioRxiv a license to display the preprint in perpetuity. It is made available under a CC-BY 4.0 International license.

References

[1] David Azriel, Samuel Davenport, and Armin Schwartzman. *Consistency of Heritability Estimation from Summary Statistics in High-Dimensional Linear Models*. arXiv, 2025. DOI: 10.48550/ARXIV.2502.11144.

[2] Brendan Bulik-Sullivan et al. "An Atlas of Genetic Correlations across Human Diseases and Traits". In: *Nature Genetics* 47.11 (Nov. 2015), pp. 1236–1241. ISSN: 1546-1718. DOI: 10.1038/ng.3406. PMID: 26414676.

[3] Brendan K. Bulik-Sullivan et al. "LD Score Regression Distinguishes Confounding from Polygenicity in Genome-Wide Association Studies". In: *Nature Genetics* 47.3 (Mar. 2015), pp. 291–295. ISSN: 1546-1718. DOI: 10.1038/ng.3211. PMID: 25642630.

[4] Brendan K. Bulik-Sullivan et al. *LD Score Regression Distinguishes Confounding from Polygenicity in Genome-Wide Association Studies Supplementary Note*. Nature Genetics, Mar. 2015.

[5] Haw-ren Fang and Dianne P. O'Leary. "Modified Cholesky Algorithms: A Catalog with New Approaches". In: *Mathematical Programming* 115.2 (Oct. 2008), pp. 319–349. ISSN: 0025-5610, 1436-4646. DOI: 10.1007/s10107-007-0177-6.

[6] *FAQ Bulik/Ldsc Wiki*. URL: https://github.com/bulik/ldsc/wiki/FAQ.

[7] Hilary K. Finucane et al. "Partitioning Heritability by Functional Annotation Using Genome-Wide Association Summary Statistics". In: *Nature Genetics* 47.11 (Nov. 2015), pp. 1228–1235. ISSN: 1546-1718. DOI: 10.1038/ng.3404. PMID: 26414678.

[8] K. B. Petersen and M. S. Pedersen. *The Matrix Cookbook*. Technical University of Denmark, Nov. 2012. URL: http://www2.compute.dtu.dk/pubdb/pubs/3274-full.html.

[9] Florian Privé et al. "Identifying and Correcting for Misspecifications in GWAS Summary Statistics and Polygenic Scores". In: *HGG advances* 3.4 (Oct. 2022), p. 100136. ISSN: 2666-2477. DOI: 10.1016/j.xhgg.2022.100136. PMID: 36105883.

[10] Armin Schwartzman et al. "A SIMPLE, CONSISTENT ESTIMATOR OF SNP HERITABILITY FROM GENOME-WIDE ASSOCIATION STUDIES". In: *The Annals of Applied Statistics* 13.4 (Dec. 2019), pp. 2509–2538. ISSN: 1932-6157. DOI: 10.1214/19-aoas1291. PMID: 38222269.

[11] Xinran Wang et al. "Twas_sim, a Python-based Tool for Simulation and Power Analysis of Transcriptome-Wide Association Analysis". In: *Bioinformatics (Oxford, England)* 39.5 (May 2023), btad288. ISSN: 1367-4811. DOI: 10.1093/bioinformatics/btad288. PMID: 37099718.

[12] Jian Yang et al. "GCTA: A Tool for Genome-Wide Complex Trait Analysis". In: *American Journal of Human Genetics* 88.1 (Jan. 2011), pp. 76–82. ISSN: 1537-6605. DOI: 10.1016/j.ajhg.2010.11.011. PMID: 21167468.

[13] Ping Yin and Xitao Fan. "Estimating R^2 Shrinkage in Multiple Regression: A Comparison of Different Analytical Methods". In: *The Journal of Experimental Education* 69.2 (Jan. 2001), pp. 203–224. ISSN: 0022-0973, 1940-0683. DOI: 10.1080/00220970109600656. URL: http://www.tandfonline.com/doi/abs/10.1080/00220970109600656 (visited on 01/16/2026).
